# A 62K genic-SNP chip array for genetic studies and breeding applications in pigeonpea (*Cajanus cajan* L. Millsp.)

**DOI:** 10.1038/s41598-020-61889-0

**Published:** 2020-03-18

**Authors:** Sangeeta Singh, Ajay K. Mahato, Pawan K. Jayaswal, Nisha Singh, Meenakshi Dheer, Preeti Goel, Ranjeet S. Raje, Jeshima K. Yasin, Rohini Sreevathsa, Vandna Rai, Kishor Gaikwad, Nagendra K. Singh

**Affiliations:** 1 ICAR-National Institute for Plant Biotechnology, Pusa Campus, New Delhi, 110012 India; 2 Division of Genetics, ICAR-Indian Agricultural Research Institute, New Delhi, 110012 India; 3 ICAR-National Bureau of Plant Genetic Resources Pusa Campus, New Delhi, 110012 India

**Keywords:** Functional genomics, Genetics, Genomics

## Abstract

Pigeonpea is the second most important pulse legume crop for food and nutritional security of South Asia that requires accelerated breeding using high throughput genomic tools. Single nucleotide polymorphisms (SNPs) are highly suitable markers for this purpose because of their bi-allelic nature, reproducibility and high abundance in the genome. Here we report on development and use of a pigeonpea 62 K SNP chip array ‘CcSNPnks’ for Affymetrix GeneTitan^®^ platform. The array was designed after filtering 645,662 genic-SNPs identified by re-sequencing of 45 diverse genotypes and has 62,053 SNPs from 9629 genes belonging to five different categories, including 4314 single-copy genes unique to pigeonpea, 4328 single-copy genes conserved between soybean and pigeonpea, 156 homologs of agronomically important cloned genes, 746 disease resistance and defense response genes and 85 multi-copy genes of pigeonpea. This fully genic chip has 28.94% exonic, 33.04% intronic, 27.56% 5′UTR and 10.46% 3′UTR SNPs and incorporates multiple SNPs per gene allowing gene haplotype network analysis. It was used successfully for the analysis of genetic diversity and population structure of 95 pigeonpea varieties and high resolution mapping of 11 yield related QTLs for number of branches, pod bearing length and number of seeds per pod in a biparental RIL population. As an accurate high-density genotyping tool, ‘CcSNPnks’ chip array will be useful for high resolution fingerprinting, QTL mapping and genome wide as well as gene-based association studies in pigeonpea.

## Introduction

Pigeonpea (*Cajanus cajan* L. Millsp.) despite being the second most important pulse crop and an important source of dietary protein in South Asia, faces serious challenges in terms of its productivity because of inadequate availability of seeds of improved varieties and various environmental stresses including pod borer (*Helicoverpa armigera*), pod fly (*Melanoagromyza obtusa*), spotted borer (*Maruca vitrata*), Fusarium wilt, sterility mosaic disease and water logging at early establishment stage. Other limitations of the extant pigeonpea varieties are their long duration, tall stature, low harvest index, spreading plant type and non-synchronous maturity, which make it difficult to manage the crop through mechanical operations. Hence, there is an urgent need to develop varieties which can overcome these limitations. The gap was fulfilled to some extent with the publication of the first draft genome of pigeonpea variety Asha^1^ but there is need to develop a high quality reference genome of pigeonpea to facilitate efficient gene discovery and molecular breeding. The SSR markers have also been developed for pigeonpea but the level of polymorphisms is very low^2^. In recent years molecular linkage maps and QTLs for important agronomic traits have been reported but more SNP markers and high-density linkage maps are required^3–5^. Establishing high throughput genotyping assays are important for gene discovery and molecular breeding in pigeonpea. High-density SNP arrays and genotyping by sequencing (GBS) have come as attractive genotyping tools. GBS using next generation sequencing can unravel unknown sequence information but its experimental operation and data analysis are beyond the reach of average breeders. In contrast high throughput genotyping arrays can be used to genotype large number of samples within a short time and the data analysis is much simpler. Several medium-density arrays have been developed for genetic analysis and breeding applications in rice^6–10^. Similarly, high-density chip arrays have been developed for rice^11–14^, sunflower^15^, soybean^16^, oil palm^17^, maize^18,19^, wheat^20,21^ and pigeonpea^22^ as well as animal species including chicken^23^, cattle^24^ and human^25^. However, all these arrays incorporate genome wide mostly intergenic SNPs and single SNP per locus, except for the 50 K genic-SNP chip of rice which is based on single-copy (SC) genes^14^. The available pigeonpea SNP array by Saxena *et al*. (2018) is also based largely (70.53%) on intergenic regions of the genome. Only 5166 of the total 56,512 SNPs in this chip belong to exonic regions^22^. The SC genes based SNP array of rice has proven efficient for studying evolutionary relationships, haplotype analysis and QTL mapping of complex traits in rice^14^.

Realizing the tremendous potential of SC genes based arrays in crop genetics and breeding, we identified such SNPs in pigeonpea by re-sequencing of 45 diverse varieties for development of a 62 K genic-SNP chip for Affymetrix GeneTitan® platform. Here we report on design, validation and use of this pigeonpea 62 K Axiom genic-SNP chip array named ‘CcSNPnks’ (for *Cajanus cajan* Single Nucleotide Polymorphism, Nagendra Kumar Singh). Out of total 56,888 protein-coding genes predicted from the improved draft of pigeonpea genome (AFSP02000000.2, https://www.ncbi.nlm.nih.gov/Traces/wgs/AFSP02?display=contigs)^26^ a reference set of 17,125 agronomically useful and SC genes were used in SNP discovery for the chip design. We demonstrate here the usefulness of this chip in analysis of genetic diversity, population structure, gene haplotype networks and high-resolution QTL mapping in pigeonpea. The 62 K SNP chip provides an opportunity to pigeonpea geneticist and breeders for identification of novel QTLs for yield, nutritional quality and resistance to various environmental stresses using bi-parental mapping population and association mapping. The chip array can also be used for background selection in marker-assisted backcross breeding for development of new pigeonpea varieties in a way similar to rice^14^.

## Results

### Design and validation of pigeonpea 62 K genic-SNP chip

**Figure 1:**
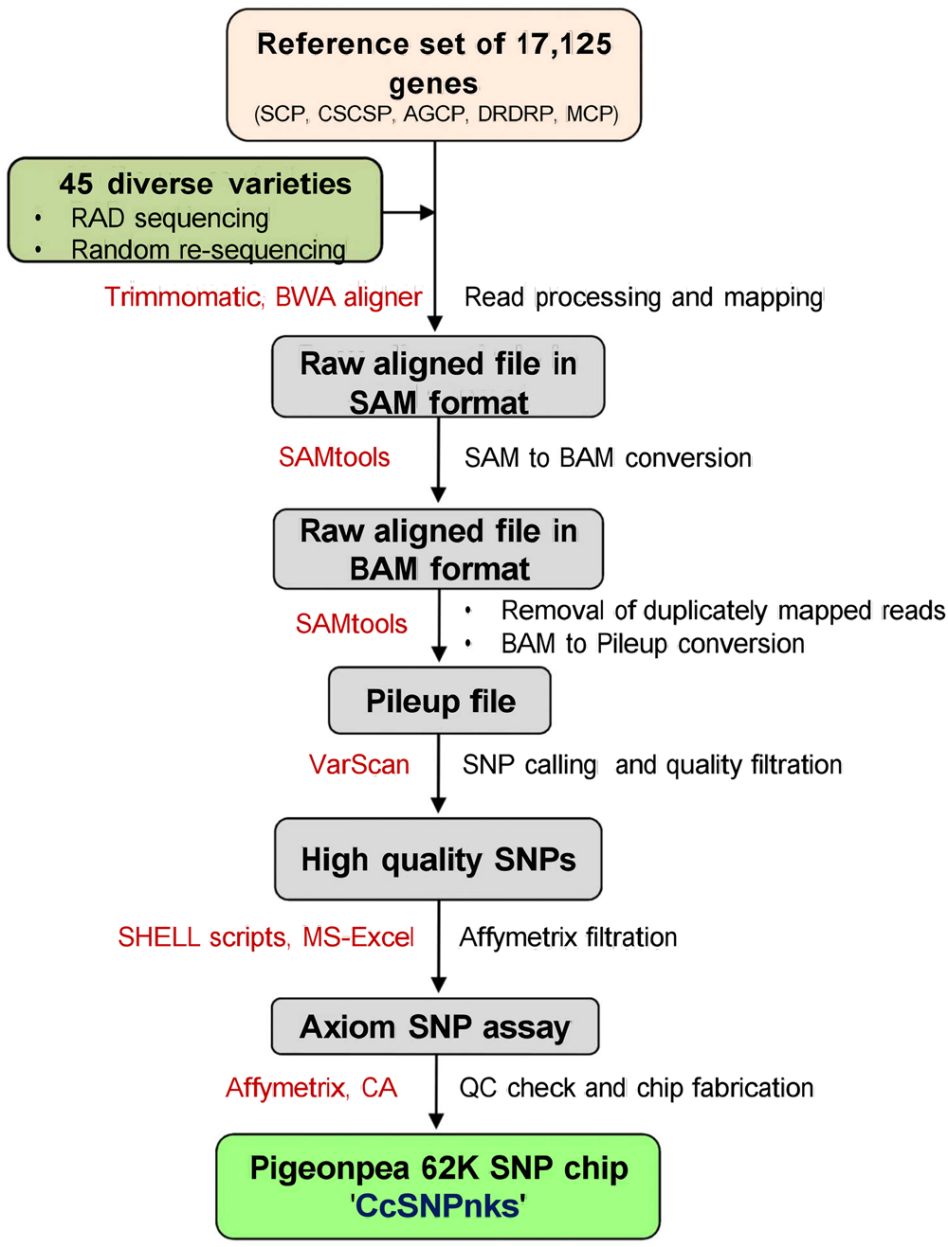
Flow diagram of the pigeonpea 62 K SNP chip design pipeline from re-sequencing till chip fabrication. Red texts on the left side of the arrows are software used and the right side black texts are the processes. Gray shaded boxes show inputs and outputs at different steps.

The 62 K genic-SNP chip described here was designed using an in-house semi-automated pipeline (Fig. 1). For SNP discovery by re-sequencing of 45 diverse pigeonpea varieties (Supplementary Table S1), initially a reduced representation library of EcoRI restriction site-associated DNA (RAD) fragments was sequenced on Illumina HiSeq platform to produce 719,993,266 high-quality sequence reads with average read length of 125 bp, generating 107.95 Gbp of sequence data that provided about 44X sequence coverage around the EcoRI restriction sites in the genome of each variety. However, alignment of sequence reads was limited to the regions flanking the EcoRI sites, which drastically reduced the chance of finding multiple SNPs per gene required for gene haplotype analysis. Therefore, we further sequenced six pools of up to eight of these varieties with equal DNA concentrations and making sequencing libraries of randomly sheared DNA fragments to produce 1,021,072,270 high quality sequence reads with average read length of 150 bp and total 127.36 Gbp of sequence data that provided 3.2X sequence coverage for entire genome of each variety. Details of re-sequencing data generated in this study are provided in Supplementary Table S2. The raw Illumina sequence reads of individual genotypes and six pools of 6–8 genotypes each utilized for SNP discovery have been submitted to the NCBI SRA database (BioProject No. PRJNA579901). Alignment of all sequence reads from the two data sets to 17,125 largely SC reference genes identified 645,662 high quality SNPs with an overall SNP density of 18.6 SNPs/Kbp (Table 1). The 17,125 reference genes belonged to five different categories: (i) 10,064 SC genes unique to pigeonpea (SCP), (ii) 5899 SC genes conserved between soybean and pigeonpea (CSCSP), (iii) 192 homologs of agronomically important cloned genes in pigeonpea (AGCP), (iv) 874 disease resistance and defense response genes in pigeonpea (DRDRP), and (v) 96 multi-copy genes in pigeonpea (MCP) used as control (Table 1). Average SNP density in these genes ranged from 6.9/Kbp for the highly conserved CSCSP genes to 111.8/Kbp for the MCP genes, whereas average number of SNPs per gene ranged from 22.1 for CSCSP genes to 234.1 for MCP genes because of the size difference in the genes.

**Table 1:** Number and percentage of genes and SNPs identifed in the reference set and finally incorporated in the 62 K Axiom SNP chip ‘CcSNPnks’ for Affymetrix platform.

Sr. no.	Gene category	No. of reference genes	Total gene length (bp)	No. of SNPs identified	SNP density per Kbp	Average gene size (bp)	No. of SNPs per gene	No. of genes in the chip	Percentage of genes in the chip	No. of SNPs in the chip	No. of SNPs per gene
1	SCP	10,064	12,218,733	430,380	35.2	1214	42.8	4314	44.80	24,537	5.7
2	CSCSP	5899	18,726,973	130,439	6.9	3175	22.1	4328	44.95	27,291	6.3
3	AGCP	192	617,039	13,979	22.7	3214	72.8	156	1.62	1536	9.9
4	DRDRP	874	2,907,123	48,387	16.6	3326	55.4	746	7.75	8090	10.8
5	MCP	96	201,088	22,477	111.8	2095	234.1	85	0.88	599	7.1
	**Total**	**17,125**	**34,670,956**	**645,662**	**18.6**	**2024**	**37.7**	**9629**	**100**	**62,053**	**6.4**

SCP = Single-copy genes unique to pigeonpea; CSCSP = Conserved single-copy genes between soyabean and pigeonpea, AGCP = Homologs of agronomically important cloned genes in pigeonpea; DRDRP = Disease resistance and defense response genes in pigeonpea; MCP = Multi-copy pigeonpea genes.

After filtering the 645,662 SNPs in 17,125 genes using Affymetrix filters for Axiom assay design 62,945 SNPs in 9648 genes were found suitable and sent to Affymetrix for chip production. Before chip production, *in silico* validation was done through a preliminary screening of the designed SNP assays for their p-convert values using Affymetrix power tool (APT) AxiomGTv1 algorithm in order to ensure a high-quality array^27^. For each SNP, both forward and reverse probes were assigned with p-convert values, derived from a random forest model to predict the probability of SNP conversion on the array. This model is based on factors including probe sequence, binding energy and expected degree of non-specific hybridization to multiple genomic regions. The SNP probes having high p-convert values are expected to convert on the SNP array with a high probability. After QC check 62,053 (62 K) SNPs in 9629 genes represented by 71,816 probes with p-convert values of >0.50 were included in the chip, showing very high conversion of our designed Axiom assays into the final chip. The 62 K SNP chip array named ‘CcSNPnks’ includes 4314 SCP, 4328 CSCSP, 156 AGCP, 746 DRDRP and 85 MCP genes (Table 1). Of the total 62,053 SNPs included in the chip, 24,537 (39.54%) were for SCP, 27,291 (43.98%) for CSCSP, 1,536 (2.47%) for AGCP, 8090 (13.03%) for DRDRP and 599 (0.96%) for MCP genes (Table 1). Overall, 9.6% of the initial 645,662 SNPs and 56.2 percent of the 17,125 reference genes were incorporated in the chip; a large proportion of the SNPs were dropped during various filtration steps of the assay design. Surprisingly, there was low 42.9% inclusion of the SCP genes in the chip as compared to 74.2–85.5% of genes from the other four categories were included.

**Figure 2:**
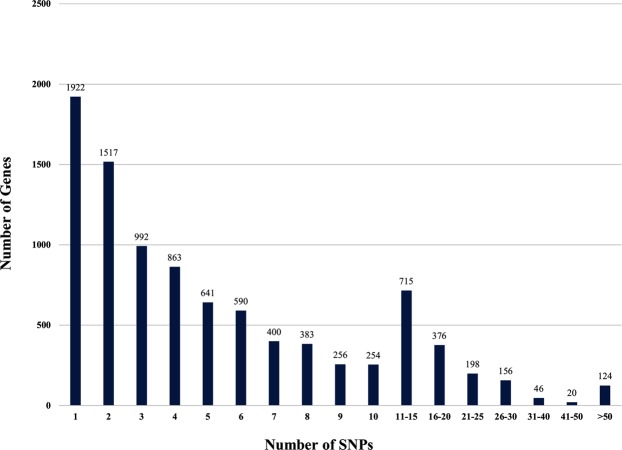
Frequency distribution plot representing number of SNPs per gene in the pigeonpea 62 K SNP chip incorporating 9,629 genes.

**Figure 3:**
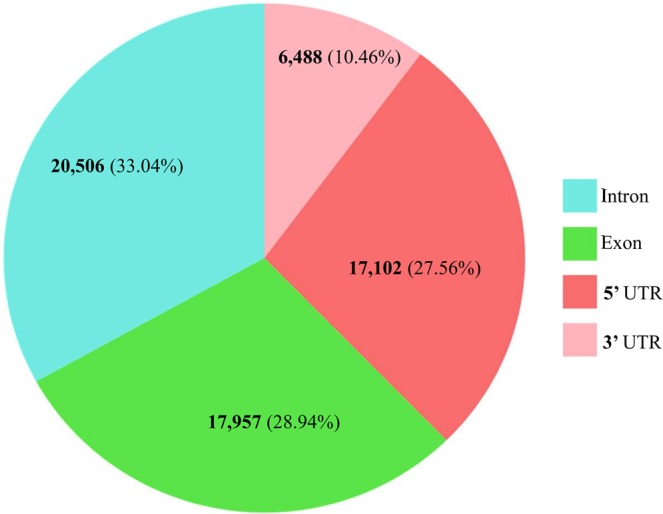
Frequency distribution and proportion of 62,053 SNPs of the pigeonpea 62 K SNP array in exonic, intronic, 5′UTR and 3′UTR regions of the 9629 genes.

This predominantly SC genes based chip array has 4314 SCP genes with an average of 5.7 SNPs per gene and 4328 CSCSP genes with an average of 6.3 SNPs per gene (Table 1). Further, it has 156 AGCP genes with an average of 9.9 SNPs per gene and 746 DRDRP genes with an average of 10.8 SNPs per gene. The 85 MCP genes included in chip have an average of 7.1 SNPs per gene, which was the lowest inclusion rate of only 3.6% of the average 234.1 SNPs in the MCP genes. The exclusion of SNPs from the chip was directly related to SNP density in the genes likely due to interference from the neighboring SNPs in the assay. Thus, the maximum inclusion of 28.5% SNPs was for the CSCSP genes, which have the lowest SNP density of 6.9 SNPs/Kbp. The complete array information of this 62 K genic-SNP chip ‘CcSNPnks’ including probe sequence, Affymetrix probes Id and NIPB SNP Id is provided in Supplementary Table S3. Despite our efforts to include multiple SNPs per gene, there were 1922 genes on the chip with single SNP and further 1517 genes with only two SNPs per gene. There was an inverse relationship between the number of SNPs per gene and the frequency of genes on the chip (Fig. 2). As for the distribution of 62,053 SNPs in different parts of the genes, 17,957 (28.94%) SNPs were present in exons, 20,506 (33.04%) in introns, 17,102 (27.56%) in 5′UTR and 6488 (10.46%) 3′UTR (Fig. 3).

Performance of the chip array was evaluated by genotyping a set of 95 diverse pigeonpea varieties on integrated Affymetrix GeneTitan® platform. Analysis of data using Axiom Analysis Suite showed all the samples passing development quality check (DQC) with a high cut-off value of 0.92 against the recommended 0.82. Actually the maximum DQC value was 0.99 with an average of 0.98. Over 95% of the samples showed high genotyping call rates of >95.5%, with an average call rate of 99.27% (Supplementary Table S4). Thus, genotyping results of 95 varieties validated the chip performance with both high sample success rate and genotyping call rates. Across genotypes, 56,468 (91%) of the 62,053 SNPs showed high call rates of >95.5%. The chip has one of the highest sample success rates of 99.7% among the existing Affymetrix Axiom SNP arrays, with minimal possibility of cross-hybridization of probes because of its SC gene based assay design. The two alleles of almost all the SNPs were clearly separated and there was easy discrimination between homozygous and heterozygous loci (Supplementary Fig. 1). Further, there was no need of imputation of genotyping data for haplotype analysis because of the high SNP call rates. Chip performance was further examined in duplicate samples of variety Pusa 2001 showing 99.9% array reproducibility. A comparison of SNP call rates among the four categories of genes showed that, CSCSP and SCP genes have the highest proportions of 91.4% and 91.0% SNPs with call rates of >95.5%, respectively. AGCP genes also showed a high 90.8% of the SNPs with call rates >95.5%, but MCP genes showed the lowest proportion of 86.8% of SNPs having call rates of >95.5%. Due to bi-allelic nature of the assays, 62 K SNP chip data unambiguously recorded the level of heterozygosity in the 95 varieties. The heterozygosity ranged between 4.89% in T-95-4 to 17.70% in Vipula with an average of 9.79%, and is likely due to often cross-pollinated nature of the species (Supplementary Table S4).

### Genetic diversity and population structure of the pigeonpea varieties

**Figure 4:**
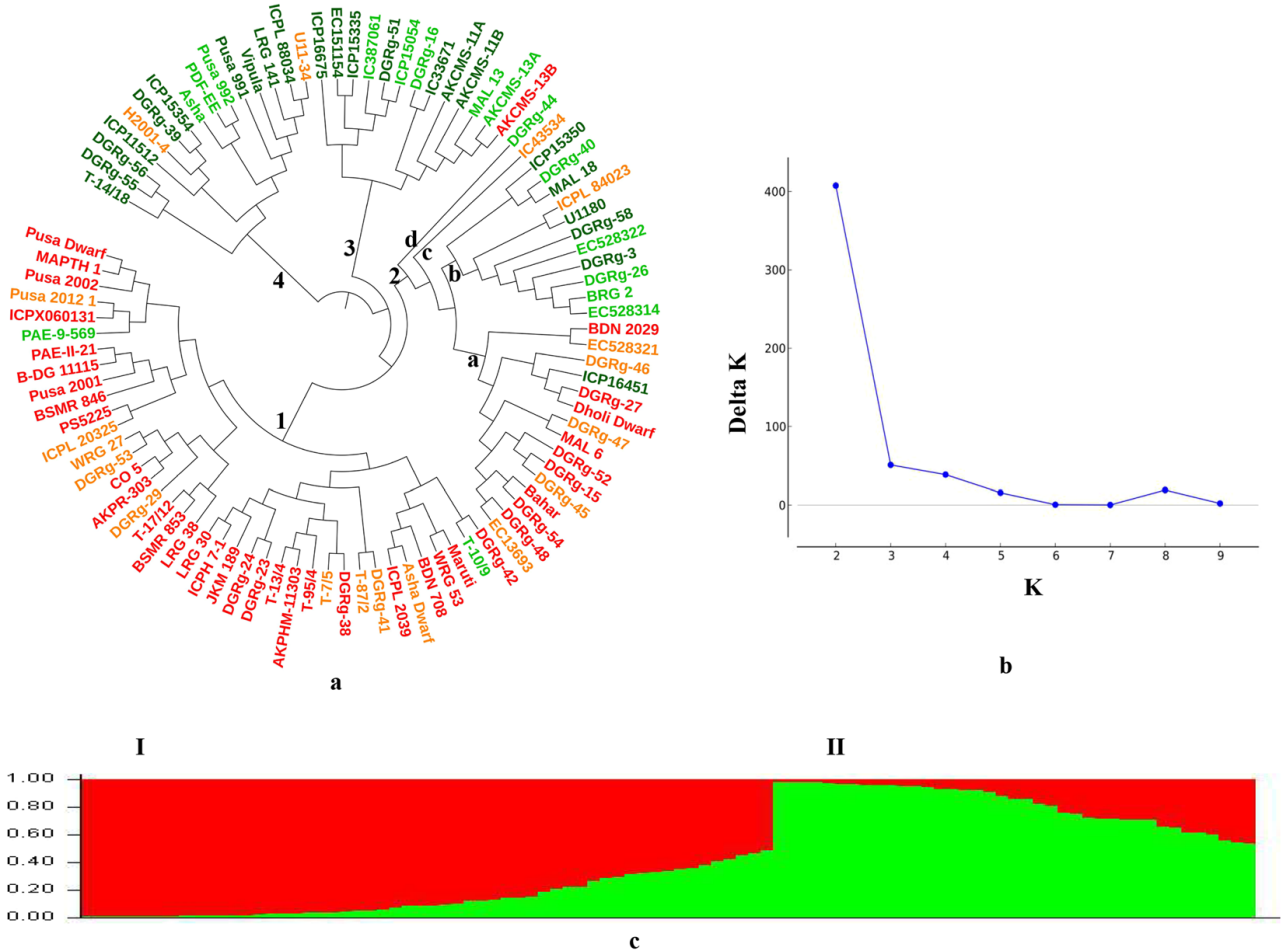
Diversity tree and population structure of 95 pigeonpea varieties: **(a)** Haplotype-based neighbor-joining tree using 30,426 SNPs with 100% call rates from the 62 K SNP chip data. Variety names are coloured according to their STRUCTURE sub-populations with lighter shades representing admixture types (Table S3). **(b)** Delta K vs. K plot of STRUCTURE analysis using 33 genome wide unlinked SNPs, showing peak at K = 2. **(c)** Population structure bar chart of the 95 pigeonpea varieties showing two sub-populations.

The genotyping data from 62 K SNP chip was used to analyze genetic diversity and population structure of 95 pigeonpea varieties. All the 95 genotypes were included in the analysis because of their high SNP call rates and 100% sample success rate (Supplementary Tables S4, S5). A NJ phylogentic tree based on 30,426 SNPs with 100 percent call rates grouped 95 varieties into four clusters (Fig. 4a). Cluster 1 comprised of 39 mostly early and medium duration varieties including CO 5, Pusa Dwarf, Pusa 2001, BSMR 853, BDN 708, DG(Rg)-23, DG(Rg)-24 and Maruti. Cluster 2 was the most heterogeneous one and comprised of 28 varieties of different maturity groups including long duration Bahar, MAL18, Dholi Dwarf, medium duration ICPL84023, BDN 2029, BRG 2 and. Early duration DG(Rg)-26, EC13693. Cluster 3 comprised of 13 mostly medium and late duration varieties including MAL 13, AKCMS 11A, AKCMS 13A, DG(Rg)-51 and ICP 15054. Cluster 4 comprised of 15 mostly early to medium duration varieties including Asha, H 2001-4, Pusa 991, Pusa 992, ICP 15354 and Vipula. However, these grouping were not strictly according to maturity groups as there were many exceptions. Baysean model based population structure of these genotypes was also analysed using a subset of only 33 genome wide unlinked SNPs with minor allele frequencies of ≥0.30 (Supplementary Tables S6, S7). The maximum delta K was reached at K = 2 and two sub-populations were inferred using Structure Harvester software (Fig. 4b). On the basis of population structure analysis 56 of the 95 varieties belonged to sub-population I and 39 varieties belonged to sub-population II (Fig. 4c). Population structure analysis further resolved these genotypes into pure and admixture types, considering accessions with Fst values of ≥0.80 as pure and those with Fst values below 0.80 as admixture type. Thus, 38 genotypes were of pure type and 18 were of admixture type in sub-population I. Similarly, 23 genotypes were of pure type and 16 were of admixture type in sub-population II. Admixture types are shown with lighter shades in Fig. 4a and Supplementary Table S7.

There was high correspondence between NJ phylogenetic tree clusters based on 30,426 SNPs and Structure sub-populations based on 33 unlinked SNPs (Fig. 4a). The NJ clusters 1 and 2a comprised of varieties largely belonging to sub-population I with exceptions of only three varieties namely PAE-9-569, T-10/9 and ICP 16451. Similarly, NJ clusters 2bcd, 3 and 4 comprised largely of varieties belonging to sub-population II with exceptions of five varieties namely ICPL 84023, IC 43534, AKCMS 13B, U-1134 and H2001-4. Six of the eight exceptions were admixture types of the two sub-populations, only two exceptions viz. ICP 16451 and AKCMS 13B were of pure types with Fst values of ≥0.80 (darker color shading in Fig. 4a, Supplementary Table S7). This showed that Bayesian model based population structure analysis using limited number of genome wide unlinked SNP markers is efficient for grouping the varieties according to their ancestry.

### Analysis of gene haplotype networks

A unique feature of the 62 K SNP chip described here is that it incorporates multiple SNPs for 7709 of the 9631 genes with an average of 6.4 SNPs per gene, which allowed haplotype network analysis for these genes (Table 1). However, there was an inverse relationship between the frequency of genes and number of SNPs per gene (Fig. 2). Haplotype networks were analyzed for eight selected genes in the 95 varieties, taking two genes each from SCP, CSCSP, AGCP and DRDRP categories with the highest number of SNPs in their category, annotated functions and hundred percent genotyping call rates (Table 2).

**Table 2:** Number of haplotypes (alleles) and haplotype diversity for eight selected genes with high number of SNPs based on haplotype network analysis of 95 diverse pigeonpea varieties.

Sr. no.	Gene Id.	Gene Annotation	No. of SNPs	No. of haplotypes	Haplotype diversity
1	SCP-4567	Exosome complex exonuclease	80	5	0.1418
2	SCP-10435	Superoxide dismutase like isoform	89	9	0.3019
3	CSCSP-4864	DNA primase large subunit	73	5	0.1225
4	CSCSP-11428	Feruloyl esterase A	83	15	0.6658
5	AGCP-21	LysM domain containing receptor kinase	128	12	0.3418
6	AGCP-178	Aldehyde dehydrogenase	108	15	0.4242
7	DRDRP-234	Serine threonine kinase	116	14	0.7380
8	DRDRP-601	TMV resistance N-like	104	20	0.5158

**Figure 5:**
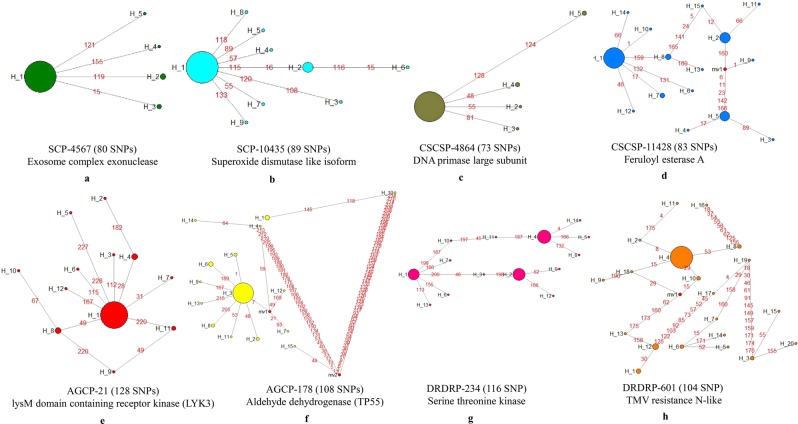
Haplotype networks of eight genes in 95 pigeonpea varieties analyzed using 62 K SNP chip, taking two genes each from four different categories having the highest number of SNPs: **(a)** and **(b)** SCP genes, **(c)** and **(d)** CSCSP gene, **(e)** and **(f)** AGCP genes, **(g)** and **(h)** DRDRP genes. The size of circles in a haplotype network is proportional to the haplotype (allele) frequency.

Haplotype network analysis of SCP-4567 gene coding for ‘exosome complex exonuclease’ with 80 SNPs showed five haplotypes (SCP-4567-H1 to SCP-4567-H5) with a low haplotype diversity (Hd) of 0.1418 (Fig. 5a). Haplotype SCP-4567-H1 was the most predominant one present in 88 of the 95 varieties including popular varieties (Pusa 992, Asha, Maruti etc). The remaining four haplotypes were minor with SCP-4567-H2 in three varieties (CO 5, ICP 16675, ICPL 20325), CP-4567-H3 in two varieties (DG(Rg)-3, T 14/18), SCP-4567-H4 in EC 528321 and SCP-4567-H5 in PAE-II-21. SCP-4567-H1 was the ancestral haplotype from which all the other four haplotypes have evolved directly. Similarly, SCP-10435 gene coding for ‘superoxide dismutase like isoform’ with 89 SNPs has nine haplotypes (SCP-10435-H1 to SCP-10435-H9) with one major and eight minor alleles and a moderate haplotype diversity of 0.3019 (Fig. 5b, Table 2). SCP-10435-H1 was the most predominant haplotype present in 79 out of 95 vareties (including Pusa 992, Pusa 2001, AKCMS-11A, Asha etc.) and ancestral to the other eight haplotypes, seven of which have evolved directly from it while one haplotype SCP-10435-H6 has evolved through SCP-10435-H2. Among the eight minor alleles SCP-10435-H2 showed the highest frequency of presence in nine genotypes (Pusa 2012-1, AKPR 303, DG(Rg)-26, DG(Rg)-3, DG(Rg)-4, DG(Rg)-46, EC 528314, EC 528321 and U11-34), the remaining seven haplotypes were present in one variety each.

Conserved single-copy gene CSCSP-4864 coding for ‘DNA primase large subunit’ with 73 SNPs showed the lowest haplotype diversity of 0.1225 with only five haplotypes (CSCSP-4864-H1 to CSCSP-4864-H5) of which CSCSP-4864-H1 was the ancestral and most predominant haplotype present in 89 of the 95 varieties, the remaining four haplotypes all derived directly from this were present in one or two varieties each (Fig. 5c, Table 2). In contrast, the other conserved single-copy gene CSCSP-11428 coding for ‘feruloyl esterase A’ with 83 SNPs showed 15 haplotypes (CSCSP-11428-H1 to CSCSP-11428-H15) with high Hd value of 0.6658 and three major haplotypes (Fig. 5d, Table 2). The major haplotypes CSCSP-11428-H1 CSCSP-11428-H2 and CSCSP-11428-H5 were present in 52, 14 and 12 varieties, respectively. Two haplotypes were present in three or four varieties each and 10 haplotypes were present in just one variety each. There are two separate evolutionary routes to the 15 haplotypes of CSCSP-11428 gene, one was directly from the major ancestral haplotype CSCSP-11428-H1 to six minor haplotypes and the other one through haplotype CSCSP-11428-H8.

Agronomically important gene AGCP-21, coding for ‘lysM domain containing receptor kinase, LYK3’ with 128 SNPs, showed 12 haplotypes (AGCP-21-H1 to AGCP-21-H12) and moderate haplotype diversity of 0.3418 (Fig. 5e, Table 2). Of the twelve haplotypes, AGCP-21-H1 was the major one present in 77 of the 95 varieties; remaining 11 haplotypes were present in one to four varieties each. Haplotypes AGCP-21-H4 and AGCP-21-H8 were present in four varieties each, AGCP-21-H11 in two varieties and the remaining eight haplotypes in one variety each. Three haplotypes, AGCP-21-H2, AGCP-21-H9, and AGCP-21-H10 were indirectly derived from AGCP-21-H1 but their origin followed two independent routes, one through AGCP-21-H11 and another one through AGCP-21-H8 (Fig. 5e). There were 15 haplotypes and moderate haplotype diversity of 0.4242 for AGCP-178 gene coding for ‘aldehyde dehydrogenase,TP55’ with 108 SNPs (Fig. 5f, Table 2). The most predominant and ancestral haplotype AGCP-178-H3 was present in 72 of the 95 varieties with seven haplotypes derived directly from this, the remaining haplotypes have separate origin. Haplotypes AGCP-178-H4 and AGCP-178-H6 were present in four varieties each, haplotypes AGCP-178-H2 and AGCP-178-H5 in two varieties each whereas the remaining haplotypes were present in one genotypes each.

Disease resistance gene DRDRP-234 coding for a ‘serine threonine kinase’ with 116 SNPs showed 14 haplotypes and the highest haplotype diversity of 0.7380 among the eight genes analysed (Fig. 5g, Table 2). It has three major haplotypes DRDRP-234-H1, DRDRP-234-H2 and DRDRP-234-H4 present in 22, 28 and 34, varieties, respectively. The second most predominant allele DRDRP-234-H1 was the ancestral haplotype giving rise to five minor alleles, two of which were roots for further evolution of eight alleles including two major haplotypes. There were three independent routes for the evolution of DRDRP-234 haplotypes. One was directly from DRDRP-234-H1, second one was from DRDRP-234-H1 through H10 to H11 to H4 giving rise to haplotypes H5, H8 and H11, while the third route was from DRDRP-234-H1 through H3 to H2 which gave rise to haplotypes H9 and H12. Disease resistance gene DRDRP-601 coding for a ‘TMV resistance N-like’ protein with 104 SNPs showed the highest number of 20 haplotypes and a high Hd value of 0.5158 (Fig. 5h, Table 2). The most predominant haplotype DRDRP-601-H4 was present in 66 varieties, haplotypes H1, H3, H6, H8, H10 and H12 were present in 2–5 varieties, while the remaining 13 haplotypes were present in one genotype each. Haplotype network analysis showed a complex evolutionary pattern for this gene with four different nodes of haplotype diversification.

Further, we examined if genotyping data on all of the large number of SNPs in a gene was needed to get the full haplotype information by taking AGCP-21 gene with 128 SNPs as an example. For this we selected three different random sets of 10 SNPs each and one random set each of 20, 40, 60, 80 and all 128 SNPs for the haplotype analysis. The number of haplotypes for the AGCP-21 gene ranged from two to four with different sets of 10 SNPs and increased successively with increasing number of SNP to maximum 12 with all the 128 SNPs (Supplementary Fig. 2). This shows that it is necessary to have data on as many SNPs as possible to get the full haplotype information, particularly the rare alleles.

### Indentification of QTLs for yield related traits

Number of branches, pod bearing length, seeds per pod and hundred seed weight are important yield contributing traits in pigeonpea. An intraspecific RIL population derived from cross between contrasting varieties Pusa Dwarf and H2001–4 was used for mapping QTLs for some of these traits using the 62 K SNP chip. High-density linkage maps of eleven pigeonpea chromosomes were constructed with 2078 filtered SNP markers (Supplementary Fig. 3). Out of total 62,053 SNPs on the chip, 11,033 were polymorphic between the two parents. These markers were further filtered for homozygous SNP calls and chi-square test of significance for 1:1 segregation ratio to obtain 3108 markers which were subjected to map construction using JoinMap. Finally, 2078 SNP markers were unambiguopusly assigned to the eleven linkage groups of pigeonpea. The remaining markers were discarded due to significant similarity of loci. The total map length was 1100.53 cM with an average marker interval of 0.88 cM (Table 3). Map length of individual linkage groups varied from of 63.38 cM for LG8 to 191.33 cM for LG5. The average marker interval was highest for LG4 (1.45 cM) with 65 SNP markers, and lowest for LG10 (0.35 cM) with 271 SNP markers. The map lengths of the two largest linkage groups LG5 (191.33 cM) and LG3 (180.62 cM) were almost double the size of other chromosomes. To avoid any confusion with the literature the numbering of linkage groups was kept consistent with the earlier publications from our laboratory^4,5^.

**Table 3:** Summary statistics of pigeonpea linkage groups (LG1 to LG11) constructed with 2078 genic-SNP markers using Pusa Dwarf/ H2001-4 RIL mapping population.

Linkage group	No. of markers	Map length (cM)	Average marker interval (cM)*
LG1	102	71.95	1.04
LG2	127	89.49	0.80
LG3	474	180.62	0.63
LG4	65	83.80	1.45
LG5	425	191.33	0.63
LG6	147	84.93	1.02
LG7	165	90.72	0.70
LG8	160	63.38	0.45
LG9	81	82.95	1.20
LG10	271	77.88	0.35
LG11	61	83.48	1.41
**Total**	**2,078**	**1,100.53**	**0.88**

^*^Estimated on the basis of number of unique loci on each linkage group, as often multiple SNPs were present at the same locus (gene).

**Figure 6:**
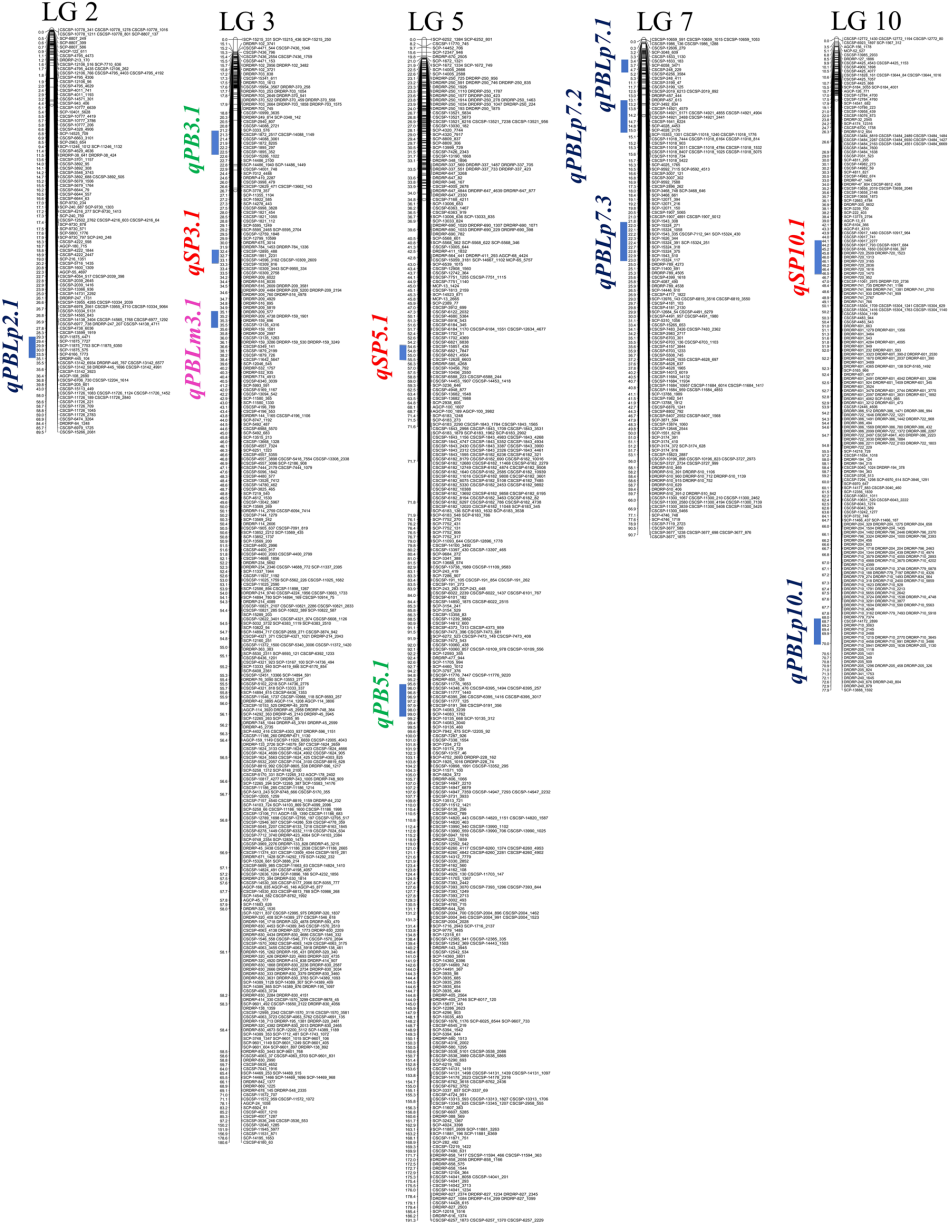
High-density linkage maps of five pigeonpea chromosomes, LG2, LG3, LG5, LG7 and LG10 developed using Pusa Dwarf/ H2001-4 RIL population, showing QTL locations for number of primary branches per plant (PB), pod bearing length on main axis (PBLm), pod bearing length on primary branches (PBLp) and number of seeds per pod (SP).

QTLs mapping for four important yield contributing traits, namely number of primary branches per plant (PB), pod bearing length on main axis (PBLm), pod bearing length on primary branches (PBLp) and number of seeds per pod (SP) were mapped based on phenotypic data from 66 RILs (Supplementary Table S8) evaluated in an augmented design with repetition of the two parental lines after every 10 RILs. Transgressive segregation with normal frequency distribution among the RILs was observed for all the four traits (Supplementary Fig. 4). Eleven major QTLs, each explaining more than 10% of the phenotypic variance were identified (Table 4). Two QTLs for number of primary branches per plant (*qPB3.1* and *qPB5.1*), each explaining 13% of the phenotypic variance were mapped on LG3 and LG5 in narrow QTL intervals of 1.34 cM and 3.04 cM, respectively. Additive effects for both the QTLs for number of primary branches were contributed by Pusa Dwarf. A major QTL for pod bearing length on the main axis (*qPBLm3.1*), contributing 12% of the phenotypic variance was identified in a 0.47 cM map interval on LG3 with additive effect contributed by the parent H2001-4. There were five QTLs for pod bearing length on the primary branches explaining phenotypic variance in the range of 13–24% and all of these except one were contributed by H2001-4 (Table 4). Only QTL *qPBLp2.1* located on LG2 was contributed by Pusa Dwarf, three QTLs namely *qPBLp7.1, qPBLp7.2* and *qPBLp7.3* contributed by H2001-4 were mapped in a closely linked genomic region on LG7, whereas an independent QTL *qPBLp10.1* also contributed by H2001-4 was mapped on LG10 (Table 4). Three major QTLs for number of seeds per pod, *qSP3.1, qSP5.1* and *qSP10.1*, contributing 17%, 64% and 47% of the phenotypic variance were mapped on LG3, LG5 and LG10, respectively. Among these, additive effect of *qSP3.1* was contributed by H2001-4 while the remaining two QTLs were contributed by Pusa Dwarf. The strongest of the three QTLs, *qSP5.1* with a LOD score of 38.4 and 64% phenotypic variance explained was located in a narrow map interval of 0.33 cM on LG5 flanked by SNP markers CSCSP6821 and CSCSP15851 (Table 4). High-density linkage maps of five pigeonpea chromosomes, LG2, LG3, LG5, LG7 and LG10 developed using RIL population and showing QTL locations for above four important yield contributing traits have been depicted in Fig. 6.

**Table 4:** QTLs for four yield related traits in pigeonpea identified in Pusa Dwarf/ H2001-4 RIL population using 62 K genic-SNP chip.

Sr. no.	Trait	QTL name	Linkage group	QTL position (cM)	QTL interval (cM)	Marker interval	LOD score	Additive effect	R^2^ (%)
1	No. of primary branches per plant	*qPB3.1*	3	21.41	1.34	SCP-3333_576 to CSCSP-1895_352	3.1	0.71	13
2	No. of primary branches per plant	*qPB5.1*	5	97.21	3.02	CSCSP-14348_476 to SCP-14083_1762	3.4	0.72	13
3	Pod bearing length on main axis	*qPBLm3.1*	3	35.51	0.47	DRDRP-209_577 to DRDRP-159_1581	3.2	−1.50	12
4	Pod bearing length on primary branch	*qPBLp2.1*	2	29.41	4.17	SCP-11875_4471 to SCP-6166_1773	6.07	1.2	21
5	Pod bearing length on primary branch	*qPBLp7.1*	7	4.71	1.57	SCP-6058_3471 to CSCSP-248_411	3.75	−1.17	24
6	Pod bearing length on primary branch	*qPBLp7.2*	7	14.71	1.88	SCP-3492_834 to SCP-15353_1301	5.92	−1.38	24
7	Pod bearing length on primary branch	*qPBLp7.3*	7	22.21	1.19	SCP-1543_308 to SCP-15324_117	4.35	−1.15	17
8	Pod bearing length on primary branch	*qPBLp10.1*	10	69.41	1.25	CSCSP-14172_2899 to DRDRP-710_2488	3.69	−1.20	13
9	No. of seeds per pod	*qSP3.1*	3	32.71	0.65	CSCSP-12895_48 to CSCSP-14596_3162	4.99	−2.6	17
10	No. of seeds per pod	*qSP5.1*	5	54.81	0.33	CSCSP-15851_436 to CSCSP-6821_4504	38.4	6.64	64
11	No. of seeds per pod	*qSP10.1*	10	46.01	2.35	CSCSP-10917_684 to DRDRP-720_1470	26.36	3.4	47

## Discussion

A major limitation in genetic dissection of complex traits in pigeonpea has been the unavailability of high-density genotyping assays that can be utilized effectively for the genetic improvement of the crop^22^. Hence, we used the improved second draft of the pigeonpea genome^26^, and re-sequencing data of 45 diverse pigeonpea varieties to develop a largely SC genes based 62 K Axiom genic-SNP chip for this purpose. Re-sequencing of 45 diverse pigeonpea varieties identified total 645,662 SNPs in a set of 17,125 reference genes with total sequence length of 34.67 Mbp. The observed SNP density of 18.6/Kbp in these genes shows that pigeonpea has one SNP every 54 bp, which is quite high contrary to the general perception of low polymorphism based on SSR markers^2,5^. The 62 K SNP chip incorporated total 62,053 SNPs in 9629 genes with an average of 6.4 SNPs per gene. Overall 9.6% of the initial 645,662 SNPs and 56.2% of the 17,125 reference genes were included in the chip; remaining SNPs were dropped during various filtration steps of the assay design viz. (i) no interfering SNP within10 bp on either side of the target SNP (ii) sequence information of 72 bp with SNP at the 36th bp position. Average SNP density in different categories of genes ranged from 6.9/Kbp in the highly conserved CSCSP genes to 111.8/Kbp in the MCP genes, whereas average number of SNPs per gene ranged from 22.1 for CSCSP genes to 234.1 for MCP genes. Surprisingly, there was very low inclusion of only 42.9% of the SCP genes in the array as compared to 74.2–85.5% of the genes from the other four categories. Likely reason for this is high SNP density of the SCP genes (35.2 SNPs/kbp) as compared to the CSCSP genes (6.9 SNPs /kbp) resulting in large number of interfering SNPs during assay design. The exclusion of SNPs was directly related to the SNP density because of interference from the neighboring SNPs in the assay. Therefore, the maximum inclusion of 28.5% SNPs was for the CSCSP genes, which have the lowest SNP density of 6.9 SNPs/Kbp (Table 1). Even though the SNP density was highest for the MCP genes, existence of very large number of average 234.1 SNPs per gene ensured that at least some of these were included in the assay. Pigeonpea was an orphan crop until the publication of its first draft genome^1^. Later genomic resources have been enhanced to a large extent but a quality finished reference genome is still awaited^1,26^. Among various types of DNA markers, SNPs are the most abundant and robust marker for the development of high-throughput genotyping assays for genetic studies and molecular breeding applications^28–31^. Conventional breeding needs expert breeders and a longer time frame of 12–15 years to develop a new improved variety because of low heritability of complex agronomic traits, whereas SNP chip arrays can be used to genotype thousands of markers in a short time for linkage mapping and association studies to identify DNA markers linked to these complex traits, which can be utilized for marker-assisted selection to reduce the breeding cycle by nearly half. SNP chip arrays are relatively simple to use in comparison to GBS that has high informatics cost and paucity of commonly genotyped loci across samples, therefore chip array is preferred over whole genome NGS based genotyping^32^. We need ultra high-density linkage map for genetic anchoring and a reference quality finishing of the available draft genomes pigeonpea^1,26,33^, which will be easy to develop using the 62 K SNP array. Also, we need trait-specific high resolution QTL mapping for foreground selection in marker-assisted breeding, which can be easily acomplished by the use of 62 K SNP chip. The 62 K SNP chip array designed here for Affymetrix Axiom®  platform is highly useful for genetic studies and molecular breeding applications in pigeonpea. It has very high sample success rate (99.7%), SNP genotyping call rate (99.9%) and assay reproducibility (99.9%) because of largely SC genes based assay design, as clearly seen by the lowest proportion of only 86.8% SNPs having call rates call rates of >95.5% in the MCP genes used as control. The MCP genes based SNPs showed 5% lower call rates thus clearly demonstrating the advantage of SC genes based array.

The CSCSP genes showed almost one-fourth the number of SNPs per gene than the SCP genes due to their highly conserved nature. Here we did a comprehensive analysis of all single-copy genes in the pigeonpea genome and found that they belonged to two different categories: (i) SCP genes, which are unique to pigeonpea, and (ii) CSCSP genes, which are conserved between pigeonpea and soybean. We have reported similar results earlier in case of rice, which also has a set of single-copy genes that are unique to rice (Singh *et al*. 2015). Hence, although conserved genes are largely of single-copy or low copy in nature, all single-copy genes are not conserved. Pigeonpea and soybean are estimated to have evolved from a common ancestor approximately 20–30 million years ago^33^. Therefore, these genes are an important resource for deciphering the evolutionary history of pigeonpea. Also, comparative analysis of SCP and MCP genes showed that SNPs in MCP genes have significantly lower call rates underlining the higher success rate of the SC genes based array. The MCP genes have very high SNP density with almost one SNP every ten base pairs of the gene, but >95% of these were dropped during assay design due to interfering SNPs.

This is the first genic-SNP genotyping chip array for pigeonpea, the available 56 K SNP chip incorporates largely intergenic SNPs^22^. The only other available high-density genic-SNP chip is SC genes based 50 K SNP chip for rice^14^. A 44 K rice SNP array comprising 44,100 SNPs from across the 12 rice chromosomes, having both genic and non-genic regions has been reported but the average SNP call rate in this chip was <92% with 4.5% of missing data^11^. Similarly, a 50 K RiceSNP50 array developed for Illumina Infinium platform has 51,478 genome-wide SNPs with only 68% coming from genic regions^13^. The utility of present 62 K SNP array was demonstrated for analysis of population structure of 95 pigeonpea genotypes inferring two sub-populations, analysis of genetic diversity, haplotype analysis for four categories of genes on the chip, construction of high-density linkage map and identification of QTLs for yield related traits in a RIL mapping population of pigeonpea (Pusa Dwarf/H2001-4). Bayesan model based population structure analysis using a subset of 33 genome wide unlinked SNPs with 100% call rates revealed two sub-populations. Genetic diversity analysis of the same set of 95 genotypes based on 30,426 SNPs with 100% call rates showed clear correspondence between NJ phylogenetic tree and population structure. Varieties in two of the four clusters of NJ phylogenetic tree showed correspondence with sub-population I, whereas those in the remaining two clusters belonged to sub-population II with very few exceptions. The comparison of clusters obtained with 62 K SNP array clearly showed that even by using a limited number of genome wide unlinked markers, varieties can be grouped according to their ancestry in an effective manner. We have also incorporated in this chip pigeonpea homologs of 156 agronomically important cloned genes in related species, which will be useful for geneticist trying to identify novel gene haplotypes linked to quality and agronomic performance of the crop. Haplotype analysis of red pericarp (*Rc1*), grain size (*GS3*), granule bound starch synthase (*GBSS1*) and soluble starch synthase (*SSS1, SSIIa, SSIIb, SSIIIa, SSIIIb, SSIVa, SSIVb*) genes of wild and cultivated rice using a similar 50 K SNP array provided clear evidence for convergent evolution of the *GBBS1* gene in wild rice and a polyphyletic orgin of the cultivated rice (Singh *et al.*, 2017)^14,34^. Similar analysis can be conducted using 62 K SNP array in pigeonpea germplasm. High-density linkage map of pigeonpea with 2078 SNP markers developed in the present study will be useful for the anchoring of pigeonpea genome, apart from the high resolution mapping of QTLs for important yield related traits. Most of these QTLs were mapped to a narrow genetic interval of less than 1.0 cM with high LOD scores, which is needed for use in marker-assisted selection. Transgressive segregation with normal frequency distribution in the RIL population was observed for all four traits, indicating that trait enahancing genes were present in both the parents that can by pyramided for yield improvement. Incorporation of 746 disease resistance and defense response genes in the array with average 10.8 SNPs per gene will be useful for pathologists and breeders in identifying genes for biotic stress resistance in pigeonpea^35^.

To date more than fifteen GBS platforms and 50 SNP arrays have been developed and utilized in 25 different plant species^36^. Despite availability of a range of multiplexing platforms, like genome wide GBS, targeted GBS, high-density arrays, medium-density arrays, low level multiplexing and single marker assays (e.g. Sequenom (Agena), KASP and Taqman platforms), high throughput genotyping platforms are not routinely used for crop breeding applications partly due to cost considerations. Cost per sample is directly proportional to the level of multiplexing which in turn depends on the exact requirement of the number of markers and samples. Despite high per sample cost high-density SNP arrays and whole genome GBS platforms have much lower costs per data point as compared to targeted GBS and medium to low multiplexing platforms^36^. High-density SNP arrays are required for specific crop breeding applications, particularly high-density fingerprinting, association studies and background selection. Thus, the 56 K SNP array developed by Saxena *et al*.^22^ and the present 62 K SNP array will be useful for pigeonpea genetic studies and breeding programme. Advantage of the 62 K SNP array is that it is a fully genic-SNP array based on single-copy genes, disease resistance genes and agronomically important genes. Another advantage is that it incorporates multiple SNPs per gene allowing scoring of haplotype (allele) information on the genes, which is necessary for allele based association mapping and selection strategies.

In conclusion the high-density 62 K genic-SNP array ‘CcSNPnks’ described here is a unique genotyping platform for pigeonpea. It has 62,053 SNPs from 9629 genes belonging to five different categories with more than 99% sample success rate and 99.9% SNP call rate. The usefulness of the array was demonstrated in population structure analysis, diversity analysis, phylogentic study, construction of high density linkage map, QTL mapping and gene haplotype analysis. Incorporation of multiple SNPs per gene is a unique feature of the array that allows gene haplotype analysis not possible with other available chips. Hence, it is an excellent platform for genetic and evolutionary studies as well as breeding applications in pigeonpea. It is an efficient tool for generating high-density linkage maps suitable for anchoring of the pigeonpea genome because of large number of markers genotyped across samples. In addition, the array would be useful for association mapping studies for correlating DNA polymorphism with phenotypic trait variation.

## Methods

### Plant material

A set of 95 diverse pigeonpea varieties used for the validation of 62 K SNP chip, diversity analysis and population structure analysis were obtained from Division of Genetics, ICAR-Indian Agricultural Research Institute, New Delhi, ICAR-National Bureau of Plant Genetic Resources, New Delhi and ICAR-National Institute for Plant Biotechnology, New Delhi (Supplementary Table S1). A subset of 45 varieties from this was re-sequenced for SNP discovery. An F_8_ recombinant inbred line (RIL) population derived from cross between Pusa Dwarf and H2001-4 used for QTL mapping was developed by Division of Genetics, ICAR-Indian Agricultural Research Institute, New Delhi.

### Selection of genes for the Axiom SNP chip array design

CDS sequences of 56,888 protein-coding genes predicted from the second draft of the pigeonpea genome were used for extracting five categories of genes for SNP mining and chip design^26^. The SC genes were identified by BLASTN search of each gene against all the 56,888 genes and recording the top five hits. Genes showing only self-match or second match with a bit score of <200 were taken as SC genes. For identification of SCP and CSCSP genes, we downloaded all the CDS sequences of three different legume species, viz. *Cicer arietinum, Glycine max and Medicago truncatula* from the GIGAd band Phytozome database (https://phytozome.jgi.doe.gov/pz/portal.html). We performed BLASTN search of all the pigeonpea SC genes against a locally created database of CDS sequences of the three legumes using pre-optimized search parameters^37^. The SCP genes did not show significant homology with any of the three species whereas CSCSP genes showed homology between pigeonpea and soybean. The AGCP genes were identified by BLASTN search of a manually curated list of cloned genes for agronomic traits in major legumes and cereals obtained through literature search against the database of 56,888 pigeonpea genes at a cutoff bit score of ≥100. In addition, 874 DRDRP genes were selected from the annotations of the first draft of the pigeonpea genome^1^. Further, we included 96 MC genes as a control to make the final set of 17,125 reference genes. In order to get information on cis-regulatory elements the reference genes were extracted in full-length along with 500 bp of upstream and 100 bp of downstream sequences from the translation start and stop codons, respectively.

### Re-sequencing, SNP discovery and array design

To identify SNPs in the reference set of genes we generated Illumina Hiseq pair-end sequence data for 45 pigeonpea genotypes (Supplementary Table S2), first using EcoR1 RAD sequencing of individually bar-coded samples for high depth of coverage, but subsequently random sheared DNA fragments in six pools of up to 8 varieties each for greater horizontal coverage of SNPs. We used an in-house pipeline for SNP mining and probe development for the Axiom SNP assays (Fig. 1). High quality SNPs were called by VarScan version 2.4.1 software using search parameters: min-coverage = 10x, min-reads = 2, min-avg-qual = 25 and min-var-freq = 0.05. The identified SNPs were filtered as per the Affemetrix requirements for the Axiom chip array, viz. (i) no interfering SNP within10 bp on either side of the target SNP (ii) SNP plex information of 72 bp with SNP at the 36th bp position. The SNP-plex information of selected SNPs was sent to Affymetrix Bioinformatics Services, San Diego for *in-silico* probe converting test, which evaluates the quality of each SNP on the basis of p-convert score using Affymetrix power tool (APT) (http://www.affymetrix.com/estore/partners_programs/programs/developer/tools/powertools.affx). Forward and reverse probes for each SNP assay were assigned p-convert values in the range of 0.0 to 1.0. Based on the p-convert values, probes were designed for each SNP and designated as ‘neutral’, ‘recommended’, ‘not recommended’ and ‘not possible‘ to help easy filtration of SNP data. Probes for the selected SNPs were synthesized directly on the array chip using Affymetrix proprietary photolithography technology. SNPs with p-convert values of <0.50 were excluded.

### Genotyping with 62 K SNP chip

Pigeonpea genomic DNA was isolated from young seedlings using CTAB method^38^, quality checked by electrophoresis in 1% agarose gel and quantified using Nano drop spectrophotometer. For target probe preparation, 20 μl of genomic DNA with concentration of 10 ng/ul was used according to Affymetrix Axiom® 2.0 Assay Manual. The samples were amplified using Target Prep Protocol QSCB1 (P/N 702990), fragmented, hybridized on the chip followed by single-base extension through DNA ligation and signal amplification. Affymetrix GeneTitan® Multi-Channel Instrument was used for staining, washing and scanning of the chip signals as per the manufacturer’s protocol (http://media.affymetrix.com/support/downloads/manuals/axiom_2_assay_auto_workflow_user_guide.pdf). SNP allele calling was done using Axiom™ Analysis Suite version 2.0 using its three workflows i.e., Best Practices, Sample QC, Genotyping and Summary (http://media.affymetrix.com/support/downloads/manuals/axiom_analysis_suite_user_guide.pdf) on the Affymetrix Gene Titan.CEL files. The Axiom Analysis Suite requires stored library files to convert.CEL files into genotype calls. SNPs with low call rate across the samples were removed and only good quality SNPs with a DQC of >0.85 and call rates of >95% were retained for further analysis.

### Analysis of genetic diversity and population structure

Genotyping data were extracted and converted to PLINK^39^ .ped and .map format and imported in the TASSEL 3.2.1 software^40^. A NJ phylogenetic tree of the 95 varieties was constructed based on 30,426 SNPs with hundred percent call rates using an improved version of neighbor-joining algorithm and visualized using iTOL software^41^. From this a subset of 33 genome wide unlinked SNPs, three from each chromosome located near the telomeres and centromere and having minor allele frequency of ≥0.3, were selected for analysis of population structure of the 95 genotypes. The model-based Bayesian approach was employed to infer population structure using STRUCTURE v. 2.3.4 software^42^. The project was run with the admixture model and correlated allele frequency using a burn in period of 50,000 and 50,000 Markov chain Monte Carlo (MCMC) replications. Five independent runs were performed with each K value ranging from 1 to 10. Evanno’s *Δ*k value was obtained by processing the STRUCTURE result using STRUCTURE HARVESTER software^43^.

### Analysis of gene haplotype network

This analysis was performed to show the utility of 62 K SNP chip in generating gene haplotypes, which is not possible with most of the available SNP chips. For this we took two genes from each of the SCP, AGCP, CSCSP and DRDRP categories present in the chip with high number of SNPs and call rates of ≥95%. Total eight genes were selected and their SNP haplotypes were generated using TASSEL 3.2.1 software^40^ in a sliding window of five nucleotides length. Haplotype networks were constructed for analysis of genealogical relationship using Network software^44^ and haplotype diversity was calculated using DnaSP software version 5.10^45^. We also analyzed the effect of taking different numbers of SNPs on the haplotype patterns of a given gene with large number of SNPs. For this we selected AGCP-21 gene with 128 SNPs and took random sets of 10, 20, 40, 60, 80 and 128 SNPs for haplotype network analysis. Three sets of 10 random SNPs were analyzed to check the effect of sampling on the haplotype.

### Mapping of QTLs for yield related traits

A set of 94 F_8_ RILs derived from cross Pusa Dwarf/ H2001-4 was used. For genotyping using 62 K SNP chip the DNA from 94 RILs along with the two parents were amplified, fragmented and hybridized on chip for SNP allele calling using Axiom™ Analysis Suite version 2.0. The genotyping data was generated for 94 RILs, alleles assigned by comparing with the two parents and linkage maps were constructed using JoinMap 4.0 software^46^. Chi-square analysis was performed on the genotyping data to test the goodness of fit against the expected 1:1 segregation ratio. The locus genotyping frequencies of JoinMap were used to identify and discard markers with aberrant segregation ratio at a cutoff P value of 0.05. Linkage groups were constructed by grouping of markers at a minimum LOD threshold of 3 to a maximum of 10 with a step of 0.5. The groups were converted to maps at LOD 3 using regression algorithm with a cutoff recombination frequency of 0.20 performing a ripple after adding 2 loci. Kosambi mapping function was used for converting recombination frequency to map distance in cM.

QTL analysis was performed for four yield related traits segregating in the RILs using WinQTL Cartographer v2.5^47^ using adjusted means of phenotypic data for the four traits from 66 RILs evaluated in an augment design. To find marker-trait associations, composite interval mapping (CIM)^48^ was performed using the ZmapQTL standard model 6 with a window size of 10 cM and 2 cM walk speed. A 1000-permutation test was performed for estimating genome-wide LOD score threshold for QTLs (P = 0.05)^49^. The additive effect and the percentage of phenotypic variation explained by each QTL were also estimated by the CIM method.

## Supplementary information

Supplementary information

Supplementary information2

Supplementary information3

Supplementary information4
