# Supplementary material for: A 62K genic-SNP chip array for genetic studies and breeding applications in pigeonpea (Cajanus cajan L. Millsp.)
Source: Sci Rep. 2020 Mar 18;10:4960. doi: 10.1038/s41598-020-61889-0 (PMC7080765; doi:10.1038/s41598-020-61889-0)
Supplement: Supplementary file 1 — Supplementary information [file 41598_2020_61889_MOESM1_ESM.docx]

**Supplementary Information**

**A 62K genic-SNP chip array for genetic studies and breeding applications in pigeonpea (*Cajanus cajan* L. Millsp*.*)**

Sangeeta Singh^1a^, Ajay K. Mahato^1a^, Pawan K. Jayaswal^1^, Nisha Singh^1^, Meenakshi Dheer^2^, Preeti Goel^2^, Ranjeet S. Raje^2^, Jeshima K. Yasin^3^, Rohini Sreevathsa^1^, Vandna Rai^1^, Kishor Gaikwad^1^ and Nagendra K. Singh^1*^

**Affiliations:**

^1^ICAR-National Institute for Plant Biotechnology, Pusa Campus, New Delhi 110012, India.

^2^Division of Genetics, ICAR-Indian Agricultural Research Institute, New Delhi, 110012, India.

^3^ICAR-National Bureau of Plant Genetic Resources PusaCampus, New Delhi, India.

^a^These authors contributed equally

**^*^ Corresponding author:** Nagendra Kumar Singh. Email: [nksingh4@gmail.com](mailto:nksingh4@gmail.com)

Tel. +91-11-2584-1186 (office)
      +91-11-2808-1376 (home)
Fax +91-11-2584-3984 (office)


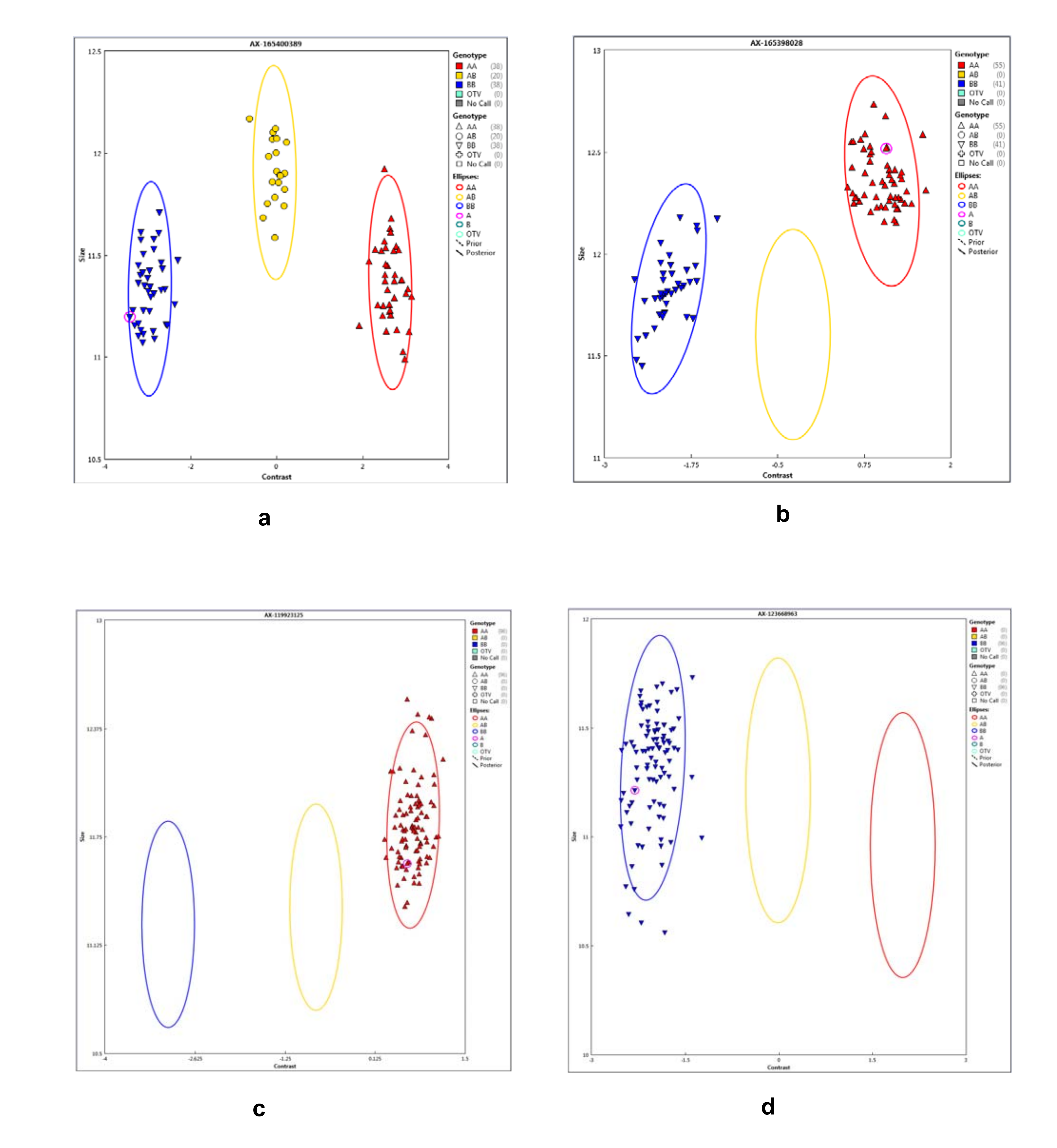


**Figure S1:** Typical SNP call patterns identified in the genotyping data on 95 pigeonpea genotypes obtained using 62K SNP chip array. **(a)** Polymorphic heterozygous SNP **(b)** Polymorphic homozygous SNP **(c, d)** Monomorphic SNPs.


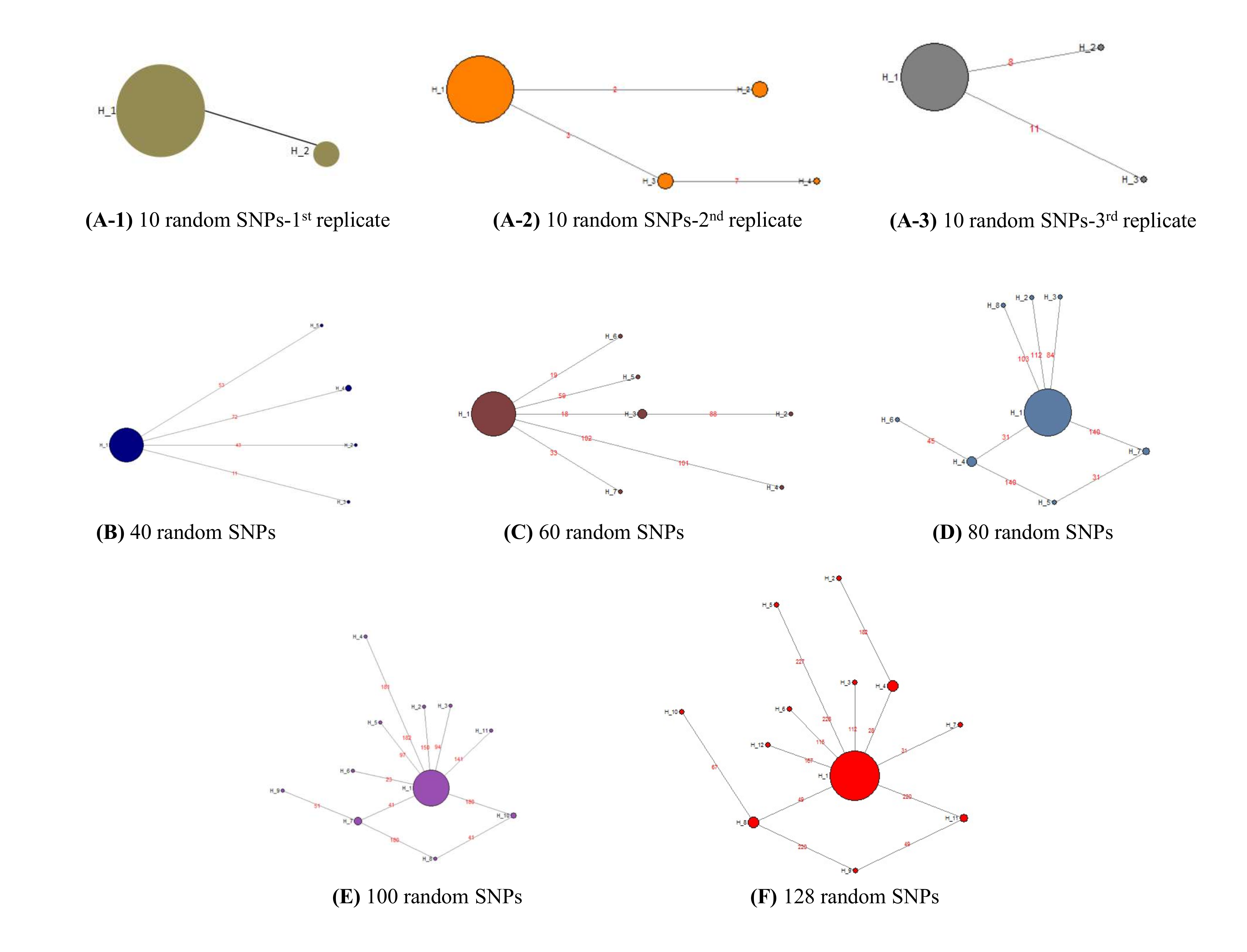


**Figure S2:** Haplotype network analysis of the pigeonpea AGCP-21 gene taking different sets of random SNPs from the total 128 SNPs in the gene based on genotyping data on 95 varieties obtained using pigeonpea 62K SNP chip. **(A-1)**10 random SNPs Ist set **(A-2)** 10 random SNPs 2^nd^ set **(A-3)** 10 random SNPs 3^rd^ seet **(B)** 40 random SNPs **(C)** 60 random SNPs **(D)** 80 random SNPs **(E)** 100 random SNPs **(F)** all 128 SNPs. The size of circles in each haplotype network is proportional to the haplotype (allele) frequency.





**Figure S3:** Linkage maps of the 11 pigeonpea chromosomes constructed with 2078 filtered polyomrphic SNP markers obtained from genotyping of Pusa Dwarf/ H2001-4 RIL population using 62K SNP array.





**Figure S4:** Phenotypic frequency distribution of yield related traits in the RIL mapping population from ‘Pusa Dawrf (**P1**)/ H2001-4 (**P2**)’ cross for: **(a)** Number of primary branches per plant, **(b)** Pod bearing length on the main axis, **(c)** Pod bearing length on primary branches, **(d)** Number of seeds per pod.

| **S. No.** | **Genotype/**  **Acc. no.** | **Germplasm/ Line Variety (year)** | **Original source** | **Seed source** | **Height group** | **Maturity group** | **Growth habit** |
| --- | --- | --- | --- | --- | --- | --- | --- |
| 1 | AKCMS-11A | Breeding line | PDKV, Akola | IARI | Tall | Medium | Indeterminate |
| 2 | AKCMS-11B | Breeding line | PDKV, Akola | IARI | Tall | Medium | Indeterminate |
| 3 | AKCMS-13A | Breeding line | PDKV, Akola | IARI | Tall | Medium | Indeterminate |
| 4 | AKCMS-13B | Breeding line | PDKV, Akola | IARI | Tall | Medium | Indeterminate |
| 5 | AKPHM-11303 | Breeding line | PDKV, Akola | IARI | Tall | Medium | Indeterminate |
| 6 | AKPR-303 | Breeding line | PDKV, Akola | IARI | Tall | Medium | Indeterminate |
| 7 | Asha Dwarf | Mutant line | BHU, Varanasi | IARI | Dwarf | Medium | Determinate |
| 8 | Asha | Variety (1993) | ICRISAT, Patancheru | IARI | Tall | Medium | Indeterminate |
| 9 | Bahar | Variety (1986) | RAU, Samastipur | IARI | Tall | Late | Indeterminate |
| 10 | BDN 2029 | Breeding line | ARS, Badnapur | IARI | Tall | Medium | Indeterminate |
| 11 | BDN 708 | Variety (2004) | ARS, Badnapur | IARI | Tall | Medium | Indeterminate |
| 12 | BRG 2 | Variety (2009) | UAS, Bengaluru | IARI | Tall | Medium | Indeterminate |
| 13 | BSMR 846 | Breeding line | ARS, Badnapur | IARI | Tall | Medium | Indeterminate |
| 14 | BSMR 853 | Variety (2001) | ARS, Badnapur | IARI | Tall | Medium | Indeterminate |
| 15 | CO 5 | Variety (1985) | TNAU, Coimbatore | IARI | Tall | Early | Indeterminate |
| 16 | Dholi Dwarf | Breeding line | RAU, Dholi | IARI | Dwarf | Late | Determinate |
| 17 | H2001-4 | Breeding line | CCSHAU, Hissar | IARI | Dwarf | Early | Indeterminate |
| 18 | ICPH 7-1 | Breeding line | ICRISAT, Patancheru | IARI | Medium | Early | Indeterminate |
| 19 | ICPL 20325 | Germplasm | ICRISAT, Patancheru | IARI | Tall | Early | Determinate |
| 20 | ICPL 2039 | Germplasm | ICRISAT, Patancheru | IARI | Medium | Medium | Determinate |
| 21 | ICPL 84023 | Germplasm | ICRISAT, Patancheru | IARI | Medium | Early | Determinate |
| 22 | ICPL 88034 | Germplasm | ICRISAT, Patancheru | IARI | Tall | Early | Indeterminate |
| 23 | ICPX060131 | Germplasm | ICRISAT, Patancheru | IARI | Tall | Early | Indeterminate |
| 24 | JKM 189 | Variety (2006) | JNKVV, Jabalpur | IARI | Tall | Medium | Indeterminate |
| 25 | LRG 41 | Breeding line | RARS, Lam | IARI | Tall | Medium | Indeterminate |
| 26 | LRG 30 | Variety (1980) | RARS, Lam | IARI | Tall | Medium | Indeterminate |
| 27 | LRG 38 | Variety (2002) | RARS, Lam | IARI | Tall | Medium | Indeterminate |
| 28 | MAL 6 | Variety (2002) | BHU, Varanasi | IARI | Tall | Late | Indeterminate |
| 29 | MAL 13 | Variety (2004) | BHU, Varanasi | IARI | Tall | Late | Indeterminate |
| 30 | MAL 18 | Breeding line | BHU, Varanasi | IARI | Tall | Late | Indeterminate |
| 31 | MAPTH 1 | Germplasm | BHU, Varanasi | IARI | Tall | Late | Indeterminate |
| 32 | Maruti | Variety (1986) | ICRISAT, Patancheru | IARI | Tall | Medium | Indeterminate |
| 33 | PS5225 | Germplasm | SKAF, Mandsore | IARI | Tall | Medium | Determinate |
| 34 | PAE-9-569 | Breeding line | IARI, New Delhi | IARI | Tall | Early | Indeterminate |
| 35 | PAE-II-21 | Breeding line | IARI, New Delhi | IARI | Tall | Early | Indeterminate |
| 36 | PDF-EE | Breeding line | IARI, New Delhi | IARI | Tall | Early | Indeterminate |
| 37 | Pusa 2001 | Variety (2006) | IARI, New Delhi | IARI | Tall | Early | Indeterminate |
| 38 | Pusa 2002 | Variety (2007) | IARI, New Delhi | IARI | Tall | Early | Indeterminate |
| 39 | Pusa 2012-1 | Breeding line | IARI, New Delhi | IARI | Tall | Early | Indeterminate |
| 40 | Pusa 991 | Variety (2005) | IARI, New Delhi | IARI | Tall | Early | Indeterminate |
| 41 | Pusa 992 | Variety (2002) | IARI, New Delhi | IARI | Tall | Early | Indeterminate |
| 42 | Pusa Dwarf | Breeding line | IARI, New Delhi | IARI | Dwarf | Early | Indeterminate |
| 43 | Vipula | Variety (2006) | MPKV, Rahuri | IARI | Tall | Medium | Indeterminate |
| 44 | WRG 53 | Variety (2009) | RARS, Warangal | IARI | Tall | Medium | Indeterminate |
| 45 | WRG 27 | Breeding line | RARS, Warangal | IARI | Medium | Medium | Indeterminate |
| 46 | B-DG 11115 | Breeding line | NBPGR, New Delhi | NBPGR | Medium | Medium | Semi erect |
| 47 | DG (Rg) 15 | Breeding line | NBPGR, New Delhi | NBPGR | Tall | Long | Compact erect |
| 48 | DG (Rg) 16 | Breeding line | NBPGR, New Delhi | NBPGR | Medium | Medium | Compact erect |
| 49 | DG(Rg)- 23 | Breeding line | NBPGR, New Delhi | NBPGR | Dwarf | Early | Compact erect |
| 50 | DG(Rg)- 24 | Breeding line | NBPGR, New Delhi | NBPGR | Dwarf | Early | Compact erect |
| 51 | DG(Rg)- 26 | Breeding line | NBPGR, New Delhi | NBPGR | Tall | Long | Semi erect |
| 52 | DG(Rg)- 27 | Breeding line | NBPGR, New Delhi | NBPGR | Medium | Long | Compact erect |
| 53 | DG(Rg)- 29 | Breeding line | NBPGR, New Delhi | NBPGR | Dwarf | Early | Compact erect |
| 54 | DG(Rg)- 3 | Breeding line | NBPGR, New Delhi | NBPGR | Medium | Long | Semi erect |
| 55 | DG(Rg)- 38 | Breeding line | NBPGR, New Delhi | NBPGR | Medium | Early | Semi erect |
| 56 | DG(Rg)- 39 | Breeding line | NBPGR, New Delhi | NBPGR | Tall | Long | Compact erect |
| 57 | DG(Rg)- 40 | Breeding line | NBPGR, New Delhi | NBPGR | Tall | Long | Semi erect |
| 58 | DG(Rg)- 41 | Breeding line | NBPGR, New Delhi | NBPGR | Tall | Medium | Semi erect |
| 59 | DG(Rg)- 42 | Breeding line | NBPGR, New Delhi | NBPGR | Tall | Medium | Semi erect |
| 60 | DG(Rg)- 44 | Breeding line | NBPGR, New Delhi | NBPGR | Medium | Long | Semi erect |
| 61 | DG(Rg)- 45 | Breeding line | NBPGR, New Delhi | NBPGR | Tall | Long | Compact erect |
| 62 | DG(Rg)- 46 | Breeding line | NBPGR, New Delhi | NBPGR | Medium | Medium | Compact erect |
| 63 | DG(Rg)- 47 | Breeding line | NBPGR, New Delhi | NBPGR | Medium | Medium | Compact erect |
| 64 | DG(Rg)- 48 | Breeding line | NBPGR, New Delhi | NBPGR | Medium | Medium | Compact erect |
| 65 | DG(Rg)- 51 | Breeding line | NBPGR, New Delhi | NBPGR | Medium | Medium | Compact erect |
| 66 | DG(Rg)- 52 | Breeding line | NBPGR, New Delhi | NBPGR | Tall | Medium | Compact erect |
| 67 | DG(Rg)- 53 | Breeding line | NBPGR, New Delhi | NBPGR | Medium | Medium | Compact erect |
| 68 | DG(Rg)- 54 | Breeding line | NBPGR, New Delhi | NBPGR | Medium | Medium | Compact erect |
| 69 | DG(Rg)- 55 | Breeding line | NBPGR, New Delhi | NBPGR | Tall | Medium | Semi erect |
| 70 | DG(Rg)- 56 | Breeding line | NBPGR, New Delhi | NBPGR | Tall | Long | Semi erect |
| 71 | DG(Rg)- 58 | Breeding line | NBPGR, New Delhi | NBPGR | Tall | Long | Semi erect |
| 72 | EC13693 | Germplasm | NBPGR, New Delhi | NBPGR | Medium | Early | Semi erect |
| 73 | EC151154 | Germplasm | NBPGR, New Delhi | NBPGR | Medium | Early | Semi erect |
| 74 | EC528314 | Germplasm | NBPGR, New Delhi | NBPGR | Medium | Early | Semi erect |
| 75 | EC528321 | Germplasm | NBPGR, New Delhi | NBPGR | Dwarf | Medium | Compact erect |
| 76 | EC528322 | Germplasm | NBPGR, New Delhi | NBPGR | Dwarf | Medium | Compact erect |
| 77 | IC33671 | Germplasm | NBPGR, New Delhi | NBPGR | Medium | Early | Semi erect |
| 78 | IC387061 | Germplasm | NBPGR, New Delhi | NBPGR | Medium | Medium | Compact erect |
| 79 | IC43534 | Germplasm | NBPGR, New Delhi | NBPGR | Tall | Long | Semi erect |
| 80 | ICP11512 | Germplasm | ICRISAT, Patancheru | NBPGR | Tall | Long | Semi erect |
| 81 | ICP15054 | Germplasm | ICRISAT, Patancheru | NBPGR | Tall | Long | Semi erect |
| 82 | ICP15335 | Germplasm | ICRISAT, Patancheru | NBPGR | Dwarf | Early | Compact erect |
| 83 | ICP15350 | Germplasm | ICRISAT, Patancheru | NBPGR | Dwarf | Early | Compact erect |
| 84 | ICP15354 | Germplasm | ICRISAT, Patancheru | NBPGR | Dwarf | Early | Compact erect |
| 85 | ICP16451 | Germplasm | ICRISAT, Patancheru | NBPGR | Medium | Long | Compact erect |
| 86 | ICP16675 | Germplasm | ICRISAT, Patancheru | NBPGR | Tall | Early | Semi erect |
| 87 | T-10/9 | Germplasm | NBPGR, New Delhi | NBPGR | Dwarf | Early | Compact erect |
| 88 | T-13/4 | Germplasm | NBPGR, New Delhi | NBPGR | Dwarf | Early | Compact erect |
| 89 | T-14/18 | Germplasm | NBPGR, New Delhi | NBPGR | Dwarf | Early | Compact erect |
| 90 | T-17/12 | Germplasm | NBPGR, New Delhi | NBPGR | Tall | Early | Semi erect |
| 91 | T-7/5 | Germplasm | NBPGR, New Delhi | NBPGR | Dwarf | Early | Compact erect |
| 92 | T-87/2 | Germplasm | NBPGR, New Delhi | NBPGR | Tall | Early | Semi erect |
| 93 | T-95/4 | Germplasm | NBPGR, New Delhi | NBPGR | Tall | Early | Semi erect |
| 94 | U11-34 | Germplasm | NBPGR, New Delhi | NBPGR | Dwarf | Medium | Compact erect |
| 95 | U1180 | Germplasm | NBPGR, New Delhi | NBPGR | Medium | Medium | Semi erect |

**Table S1:** List of 95 diverse pigeonpea genotypes used for the development and validation of 62K SNP chip array. Serial numbers 1-45 were used for SNP discovery by re-sequencing, and all 95 genotypes were used for chip validation and population structure analysis.

| **Sr. No.** | **Variety Name** | **Raw Reads** | **Clean Reads** | **Raw Base (Gbp)** | **Clean Base (Gbp)** | **Effective Rate (%)** | **Error Rate (%)** | **Q20 (%)** | **Q30**  **(%)** | **GC Content (%)** |
| --- | --- | --- | --- | --- | --- | --- | --- | --- | --- | --- |
| 1 | AKCMS-11A | 8898823 | 8727532 | 2.225 | 2.182 | 98.08 | 0.04 | 94.75 | 90.22 | 35.92 |
| 2 | AKCMS-11B | 5063759 | 4968348 | 1.266 | 1.242 | 98.12 | 0.04 | 95.06 | 90.84 | 34.55 |
| 3 | AKCMS-13A | 5073251 | 4966064 | 1.268 | 1.242 | 97.89 | 0.04 | 94.71 | 90.20 | 34.44 |
| 4 | AKCMS-13B | 4277556 | 4173337 | 1.069 | 1.043 | 97.56 | 0.04 | 94.84 | 90.42 | 35.44 |
| 5 | AKPHM-11303 | 17627528 | 17305618 | 4.407 | 4.326 | 98.17 | 0.04 | 94.69 | 90.19 | 34.44 |
| 6 | AKPR-303 | 20077104 | 19748333 | 5.019 | 4.937 | 98.36 | 0.04 | 94.81 | 90.40 | 34.41 |
| 7 | Asha | 14482674 | 14086003 | 3.621 | 3.522 | 97.26 | 0.04 | 93.55 | 88.29 | 35.42 |
| 8 | Asha Dwarf | 10913213 | 10576214 | 2.728 | 2.644 | 96.91 | 0.04 | 93.75 | 88.65 | 34.77 |
| 9 | Bahar | 18765459 | 18485095 | 4.691 | 4.621 | 98.51 | 0.04 | 95.00 | 90.74 | 34.02 |
| 10 | BDN 2029 | 6996281 | 6835934 | 1.749 | 1.709 | 97.71 | 0.04 | 94.03 | 89.09 | 34.78 |
| 11 | BDN 708 | 17980517 | 17584693 | 4.495 | 4.396 | 97.80 | 0.04 | 94.11 | 89.22 | 34.94 |
| 12 | BRG-2 | 6147017 | 6029371 | 1.537 | 1.507 | 98.09 | 0.04 | 94.86 | 90.48 | 35.02 |
| 13 | BSMR 846 | 14735459 | 14380631 | 3.684 | 3.595 | 97.59 | 0.04 | 94.03 | 89.09 | 34.41 |
| 14 | BSMR 853 | 12689820 | 12481297 | 3.172 | 3.12 | 98.36 | 0.04 | 94.94 | 90.60 | 36.31 |
| 15 | CO 5 | 10431377 | 10241331 | 2.608 | 2.56 | 98.18 | 0.04 | 94.79 | 90.34 | 35.52 |
| 16 | Dholi Dwarf | 14068499 | 13635566 | 3.517 | 3.409 | 96.92 | 0.04 | 93.75 | 88.66 | 34.70 |
| 17 | H2001-4 | 11794782 | 11541844 | 2.949 | 2.885 | 97.86 | 0.04 | 94.16 | 89.37 | 34.42 |
| 18 | ICPH-7 | 9553487 | 9356252 | 2.388 | 2.339 | 97.94 | 0.04 | 94.12 | 89.22 | 34.81 |
| 19 | ICPL20325 | 10245720 | 10052912 | 2.561 | 2.513 | 98.12 | 0.04 | 94.29 | 89.59 | 34.42 |
| 20 | ICPL2039 | 23832189 | 23492331 | 5.958 | 5.873 | 98.57 | 0.04 | 95.32 | 91.25 | 35.25 |
| 21 | ICPL84023 | 15150861 | 14858981 | 3.788 | 3.715 | 98.07 | 0.04 | 94.22 | 89.42 | 34.64 |
| 22 | ICPL88034 | 19117972 | 18740756 | 4.779 | 4.685 | 98.03 | 0.04 | 94.20 | 89.47 | 34.64 |
| 23 | ICPX060131 | 10634136 | 10377884 | 2.659 | 2.594 | 97.59 | 0.04 | 93.69 | 88.50 | 34.50 |
| 24 | JKM-189 | 10520985 | 10348642 | 2.63 | 2.587 | 98.36 | 0.04 | 94.89 | 90.53 | 36.03 |
| 25 | LRG-141 | 7622262 | 7490628 | 1.906 | 1.873 | 98.27 | 0.04 | 94.93 | 90.62 | 35.92 |
| 26 | LRG-30 | 8896016 | 8754167 | 2.224 | 2.189 | 98.41 | 0.04 | 94.91 | 90.52 | 36.16 |
| 27 | LRG-38 | 12429717 | 12213713 | 3.107 | 3.053 | 98.26 | 0.04 | 94.75 | 90.27 | 34.37 |
| 28 | MAL 6 | 12091107 | 11888085 | 3.023 | 2.972 | 98.32 | 0.04 | 94.89 | 90.52 | 34.92 |
| 29 | MAL 13 | 12202256 | 11996701 | 3.051 | 2.999 | 98.32 | 0.04 | 94.94 | 90.59 | 35.40 |
| 30 | MAL 18 | 9112427 | 8978525 | 2.278 | 2.245 | 98.53 | 0.04 | 95.25 | 91.15 | 35.09 |
| 31 | MAPTH-1 | 15217669 | 14910867 | 3.804 | 3.728 | 97.98 | 0.04 | 94.11 | 89.25 | 34.95 |
| 32 | Maruti | 5123082 | 5026123 | 1.281 | 1.257 | 98.11 | 0.04 | 95.00 | 90.76 | 35.25 |
| 33 | P55225 | 8787348 | 8515576 | 2.197 | 2.129 | 96.91 | 0.04 | 93.74 | 88.66 | 34.50 |
| 34 | PAE-9-569 | 15129413 | 14677727 | 3.782 | 3.669 | 97.01 | 0.04 | 94.05 | 89.12 | 34.98 |
| 35 | PAE-II-21 | 11052627 | 10785152 | 2.763 | 2.696 | 97.58 | 0.04 | 93.72 | 88.53 | 34.52 |
| 36 | PDF-EE | 7615834 | 7219021 | 1.912 | 1.854 | 96.97 | 0.04 | 93.82 | 88.42 | 34.62 |
| 37 | Pusa 2001 | 10907103 | 10615776 | 2.727 | 2.654 | 97.33 | 0.04 | 93.97 | 89.02 | 35.53 |
| 38 | Pusa 2002 | 7192706 | 6985910 | 1.798 | 1.746 | 97.12 | 0.04 | 94.13 | 89.30 | 34.16 |
| 39 | Pusa 2012-1 | 5531288 | 5399859 | 1.383 | 1.35 | 97.62 | 0.04 | 94.28 | 89.58 | 33.81 |
| 40 | Pusa 991 | 14558307 | 14223123 | 3.64 | 3.556 | 97.70 | 0.04 | 93.86 | 88.86 | 35.03 |
| 41 | Pusa 992 | 11384646 | 11012017 | 2.846 | 2.753 | 96.73 | 0.04 | 93.78 | 88.66 | 35.35 |
| 42 | Pusa Dwarf | 8102521 | 7860625 | 2.026 | 1.965 | 97.01 | 0.04 | 93.86 | 88.86 | 34.53 |
| 43 | Vipula | 11104928 | 10947329 | 2.776 | 2.737 | 98.58 | 0.04 | 95.20 | 91.03 | 35.17 |
| 44 | WRG 53 | 6952863 | 6830232 | 1.738 | 1.708 | 98.24 | 0.04 | 94.92 | 90.61 | 34.09 |
| 45 | WRP 27 | 14655844 | 14410812 | 3.664 | 3.603 | 98.33 | 0.04 | 94.78 | 90.31 | 35.57 |
| 46 | Pool 1 | 62561463 | 60997426 | 18.768 | 18.665 | 97.25 | 0.04 | 95.32 | 89.02 | 35.25 |
| 47 | Pool 2 | 67206140 | 65862017 | 19.087 | 18.987 | 98.50 | 0.04 | 94.75 | 88.66 | 34.64 |
| 48 | Pool 3 | 59261371 | 58396155 | 16.949 | 16.265 | 98.36 | 0.04 | 94.05 | 90.76 | 35.17 |
| 49 | Pool 4 | 55316073 | 54403358 | 16.374 | 16.052 | 98.04 | 0.04 | 94.29 | 89.42 | 35.92 |
| 50 | Pool 5 | 68796318 | 53722970 | 19.676 | 19.159 | 97.37 | 0.04 | 94.11 | 90.62 | 34.44 |
| 51 | Pool 6 | 47756116 | 46380740 | 13.754 | 13.019 | 97.12 | 0.04 | 93.78 | 89.30 | 34.42 |

**Table S2:** Summary of Ilumina sequence reads data from EcoRI RAD sequencing of 45 individual varieties (**Sr. no. 1-45**), five pools of eight varieties each (**Sr. no. 47-50**) and one pool of five varieties (**S. no. 51**) used for SNP discovery **(NCBI Bio-project No. PRJNA579901; Study No. SRP227326; SRA Acc. No. SSR10359277-SSR10359316 and SSR 10758020-SSR10758025)**

**Table S3:** Pigeonpea 62K SNP assay information including Affymetrix Probe Id and NIPB SNP Id, containing gene number and SNP flanking DNA sequence.

**(Separate Excel file: ‘Table S3 pigeonpea 62K SNP chip probe information.xlsx’.**

| **Sr. no.** | **Sample name** | **DQC** | **Call rate (%)** | **Hetero-zygosity**  **(%)** | **Homo-zygosity**  **(%)** |
| --- | --- | --- | --- | --- | --- |
|  | AKCMS-11A | 0.96 | 99.38 | 16.23 | 83.77 |
|  | AKCMS-11B | 0.95 | 99.37 | 14.09 | 85.91 |
|  | AKCMS-13A | 0.98 | 99.80 | 12.38 | 87.62 |
|  | AKCMS-13B | 0.96 | 99.28 | 7.11 | 92.89 |
|  | AKPHM-11303 | 0.99 | 99.71 | 5.16 | 94.84 |
|  | AKPR-303 | 0.98 | 99.59 | 6.22 | 93.78 |
|  | Asha | 0.96 | 99.16 | 16.84 | 83.16 |
|  | Asha Dwarf | 0.97 | 99.06 | 5.57 | 94.43 |
|  | B-DG11115 | 0.98 | 99.73 | 9.87 | 90.13 |
|  | Bahar | 0.98 | 99.73 | 5.76 | 94.24 |
|  | BDN2029 | 0.95 | 98.07 | 11.72 | 88.28 |
|  | BDN708 | 0.98 | 99.78 | 5.36 | 94.64 |
|  | BRG-2 | 0.97 | 99.27 | 7.93 | 92.07 |
|  | BSMR-853 | 0.98 | 99.73 | 7.31 | 92.70 |
|  | BSMR846 | 0.96 | 99.32 | 11.22 | 88.78 |
|  | CO 5 | 0.98 | 99.66 | 5.85 | 94.15 |
|  | DG(Rg)-15 | 0.99 | 99.68 | 5.41 | 94.59 |
|  | DG(Rg)-16 | 0.99 | 99.71 | 11.17 | 88.83 |
|  | DG(Rg)-23 | 0.99 | 99.80 | 5.55 | 94.45 |
|  | DG(Rg)-24 | 0.97 | 94.47 | 7.00 | 93.00 |
|  | DG(Rg)-26 | 0.99 | 99.86 | 11.24 | 88.76 |
|  | DG(Rg)-27 | 0.99 | 99.04 | 13.16 | 86.84 |
|  | DG(Rg)-29 | 0.97 | 99.68 | 14.61 | 85.39 |
|  | DG(Rg)-3 | 0.99 | 99.75 | 12.90 | 87.10 |
|  | DG(Rg)-38 | 0.98 | 99.15 | 7.23 | 92.78 |
|  | DG(Rg)-39 | 0.99 | 99.75 | 14.44 | 85.56 |
|  | DG(Rg)-40 | 0.98 | 99.78 | 8.52 | 91.48 |
|  | DG(Rg)-41 | 0.99 | 99.80 | 6.73 | 93.27 |
|  | DG(Rg)-42 | 0.99 | 99.62 | 7.16 | 92.84 |
|  | DG(Rg)-44 | 0.99 | 99.75 | 10.73 | 89.28 |
|  | DG(Rg)-45 | 0.99 | 99.76 | 7.51 | 92.49 |
|  | DG(Rg)-46 | 0.98 | 99.76 | 7.16 | 92.84 |
|  | DG(Rg)-47 | 0.99 | 99.59 | 7.27 | 92.73 |
|  | DG(Rg)-48 | 0.98 | 99.65 | 5.16 | 94.84 |
|  | DG(Rg)-51 | 0.98 | 99.77 | 14.05 | 85.95 |
|  | DG(Rg)-52 | 0.98 | 99.71 | 7.50 | 92.50 |
|  | DG(Rg)-53 | 0.99 | 99.73 | 8.26 | 91.74 |
|  | DG(Rg)-54 | 0.98 | 99.64 | 6.06 | 93.94 |
|  | DG(Rg)-55 | 0.99 | 99.85 | 12.78 | 87.22 |
|  | DG(Rg)-56 | 0.99 | 99.78 | 13.14 | 86.86 |
|  | DG(Rg)-58 | 0.98 | 99.69 | 9.89 | 90.11 |
|  | Dholi Dwarf | 0.98 | 99.76 | 5.57 | 94.43 |
|  | EC13693 | 0.99 | 99.81 | 6.32 | 93.68 |
|  | EC151154 | 0.99 | 99.77 | 14.51 | 85.49 |
|  | EC528314 | 0.99 | 99.71 | 12.18 | 87.82 |
|  | EC528321 | 0.99 | 99.48 | 11.23 | 88.77 |
|  | EC528322 | 0.99 | 99.61 | 11.33 | 88.67 |
|  | H2001-4 | 0.93 | 98.67 | 14.63 | 85.37 |
|  | IC33671 | 0.98 | 98.08 | 10.79 | 89.21 |
|  | IC387061 | 0.99 | 99.83 | 10.84 | 89.16 |
|  | IC43534 | 0.98 | 99.63 | 6.84 | 93.16 |
|  | ICP11512 | 0.99 | 91.59 | 15.23 | 84.77 |
|  | ICP15054 | 0.97 | 99.72 | 11.07 | 88.93 |
|  | ICP15335 | 0.98 | 99.72 | 9.34 | 90.66 |
|  | ICP15350 | 0.98 | 99.59 | 12.47 | 87.53 |
|  | ICP15354 | 0.99 | 99.85 | 14.46 | 85.54 |
|  | ICP16451 | 0.98 | 99.41 | 14.02 | 85.98 |
|  | ICP16675 | 0.99 | 99.73 | 12.42 | 87.58 |
|  | ICPH-7 | 0.98 | 99.77 | 5.41 | 94.59 |
|  | ICPL-84023 | 0.96 | 98.82 | 10.34 | 89.66 |
|  | ICPL20325 | 0.93 | 98.15 | 13.20 | 86.80 |
|  | ICPL2039 | 0.98 | 99.66 | 5.48 | 94.52 |
|  | ICPL88034 | 0.96 | 99.63 | 13.85 | 86.15 |
|  | ICPX060131 | 0.97 | 99.70 | 6.57 | 93.44 |
|  | JKM-189 | 0.99 | 99.32 | 7.90 | 92.10 |
|  | LRG 141 | 0.98 | 99.68 | 14.01 | 85.99 |
|  | LRG 30 | 0.98 | 99.74 | 5.09 | 94.91 |
|  | LRG 38 | 0.96 | 98.35 | 10.55 | 89.45 |
|  | MAL 6 | 0.97 | 99.59 | 6.34 | 93.66 |
|  | MAL 13 | 0.99 | 99.04 | 9.19 | 90.82 |
|  | MAL 18 | 0.97 | 98.48 | 10.54 | 89.47 |
|  | MAPTH-1 | 0.99 | 99.51 | 5.75 | 94.25 |
|  | Maruti | 0.98 | 99.59 | 6.17 | 93.83 |
|  | PS-5225 | 0.98 | 99.75 | 7.07 | 92.93 |
|  | PAE-9-569 | 0.95 | 99.63 | 12.66 | 87.34 |
|  | PAE-II-21 | 0.92 | 98.22 | 13.45 | 86.56 |
|  | PDF-EE | 0.99 | 99.84 | 13.56 | 86.45 |
|  | Pusa 2001 | 0.98 | 99.74 | 7.95 | 92.05 |
|  | Pusa 2002 | 0.95 | 98.42 | 12.22 | 87.78 |
|  | Pusa 2012-1 | 0.99 | 99.89 | 11.51 | 88.49 |
|  | Pusa 991 | 0.97 | 93.26 | 15.80 | 84.20 |
|  | Pusa 992 | 0.98 | 99.76 | 8.77 | 91.23 |
|  | Pusa Dwarf | 0.99 | 99.75 | 5.42 | 94.58 |
|  | T-10-9 | 0.98 | 99.77 | 9.89 | 90.11 |
|  | T-13-4 | 0.98 | 99.70 | 5.28 | 94.72 |
|  | T-14-18 | 0.97 | 99.53 | 15.44 | 84.56 |
|  | T-17-12 | 0.99 | 99.75 | 7.08 | 92.92 |
|  | T-7-5 | 0.99 | 99.80 | 8.38 | 91.62 |
|  | T-87-2 | 0.97 | 99.60 | 8.05 | 91.95 |
|  | T-95-4 | 0.97 | 99.64 | 4.89 | 95.11 |
|  | U11-34 | 0.98 | 99.87 | 9.06 | 90.94 |
|  | U1180 | 0.98 | 98.64 | 11.86 | 88.14 |
|  | Vipula | 0.98 | 95.84 | 17.70 | 82.31 |
|  | WRG-53 | 0.99 | 99.66 | 5.81 | 94.19 |
|  | WRP-27 | 0.98 | 99.77 | 11.61 | 88.39 |
|  | **Average** | **0.98** | **99.27** | **9.79** | **90.21** |

**Table S4:** 62K SNP chip array genotyping data on 95 pigeonpea genotypes with respect to DQC, call rates and percent heterozygosity.

**Table S5:** Full genotyping data on 30,426 SNPs with 100 percent call rates on 95 pigoenpea genotypes, used for the diversity analysis and construction of NJ phylogenetic tree.

**(Separate Excel file: ‘Table S5 pigeonpea 62K SNP chip 30,426 SNP data.xlsx’.**

**Table S6:** Pigeonpea 62K SNP assay information including Affymetrix Probe Id and NIPB SNP Id, containing gene number SNP flanking DNA sequence of 33 genome wide unlinked SNPs, three on each of the 11 pigeonpea linkage used for population structure analysis.

**(Separate Excel file: ‘Table S6 pigeonpea 62K SNP chip 33 SNP data.xlsx’.**

| **S. No.** | **Genotype** | **Source of genotype** | **Fst Value** | |
| --- | --- | --- | --- | --- |
|  |  |  | **Sub-population I** | **Sub-population II** |
|  | **AKPHM-11303*** | ICAR-IARI | 0.990 | 0.010 |
|  | **AKPR-303*** | ICAR-IARI | 0.988 | 0.012 |
|  | **Bahar*** | ICAR-IARI | 0.988 | 0.012 |
|  | **ICPL2039*** | ICAR-IARI | 0.988 | 0.012 |
|  | **MAPTH-1*** | ICAR-IARI | 0.988 | 0.012 |
|  | **T-95-4** | ICAR-NBPGR | 0.988 | 0.012 |
|  | **LRG-38*** | ICAR-IARI | 0.987 | 0.013 |
|  | **MAL 6*** | ICAR-IARI | 0.986 | 0.014 |
|  | **LRG-30*** | ICAR-IARI | 0.985 | 0.015 |
|  | **Pusa Dwarf*** | ICAR-IARI | 0.985 | 0.015 |
|  | **WRG-53*** | ICAR-IARI | 0.984 | 0.016 |
|  | **ICPX060131*** | ICAR-IARI | 0.983 | 0.017 |
|  | **BDN 708*** | ICAR-IARI | 0.981 | 0.019 |
|  | **T-17-12** | ICAR-NBPGR | 0.981 | 0.019 |
|  | **DG(Rg)-38** | ICAR-NBPGR | 0.978 | 0.022 |
|  | **T-13-4** | ICAR-NBPGR | 0.971 | 0.029 |
|  | **ICPH-7*** | ICAR-IARI | 0.970 | 0.030 |
|  | **DG(Rg)-48** | ICAR-NBPGR | 0.965 | 0.035 |
|  | **Dholi Dwarf*** | ICAR-IARI | 0.963 | 0.037 |
|  | **DG(Rg)-15** | ICAR-NBPGR | 0.961 | 0.039 |
|  | **DG(Rg)-24** | ICAR-NBPGR | 0.960 | 0.040 |
|  | **BDN 2029*** | ICAR-IARI | 0.957 | 0.043 |
|  | **DG(Rg)-42** | ICAR-NBPGR | 0.950 | 0.050 |
|  | **PAE-II-21*** | ICAR-IARI | 0.943 | 0.057 |
|  | **Maruti*** | ICAR-IARI | 0.936 | 0.064 |
|  | **DG(Rg)-52** | ICAR-NBPGR | 0.928 | 0.072 |
|  | **Pusa 2002*** | ICAR-IARI | 0.913 | 0.087 |
|  | **Pusa 2001*** | ICAR-IARI | 0.912 | 0.088 |
|  | **JKM-189*** | ICAR-IARI | 0.911 | 0.089 |
|  | **DG(Rg)-27** | ICAR-NBPGR | 0.903 | 0.097 |
|  | **PS-5225*** | ICAR-IARI | 0.895 | 0.105 |
|  | **DG(Rg)-54** | ICAR-NBPGR | 0.874 | 0.126 |
|  | **CO 5*** | ICAR-IARI | 0.872 | 0.128 |
|  | **AKCMS-13B*** | ICAR-IARI | 0.866 | 0.134 |
|  | **BSMR846*** | ICAR-IARI | 0.856 | 0.144 |
|  | **DG(Rg)-23** | ICAR-NBPGR | 0.851 | 0.149 |
|  | **BSMR-853*** | ICAR-IARI | 0.844 | 0.156 |
|  | **B-DG11115** | ICAR-NBPGR | 0.810 | 0.190 |
|  | **T-7-5** | ICAR-NBPGR | 0.786 | 0.214 |
|  | **DG(Rg)-53** | ICAR-NBPGR | 0.776 | 0.224 |
|  | **DG(Rg)-47** | ICAR-NBPGR | 0.774 | 0.226 |
|  | **ICPL20325*** | ICAR-IARI | 0.730 | 0.270 |
|  | **DG(Rg)-46** | ICAR-NBPGR | 0.711 | 0.289 |
|  | **EC13693** | ICAR-NBPGR | 0.701 | 0.299 |
|  | **IC43534** | ICAR-NBPGR | 0.684 | 0.316 |
|  | **Asha Dwarf*** | ICAR-NRCPB | 0.676 | 0.324 |
|  | **DG(Rg)-45** | ICAR-NBPGR | 0.666 | 0.334 |
|  | **T-87-2** | ICAR-NBPGR | 0.662 | 0.338 |
|  | **EC528321** | ICAR-NBPGR | 0.643 | 0.357 |
|  | **DG(Rg)-41** | ICAR-NBPGR | 0.642 | 0.358 |
|  | **Pusa 2012-1*** | ICAR-IARI | 0.621 | 0.379 |
|  | **U11-34** | ICAR-NBPGR | 0.587 | 0.413 |
|  | **ICPL-84023*** | ICAR-IARI | 0.578 | 0.422 |
|  | **WRP 27*** | ICAR-IARI | 0.545 | 0.455 |
|  | **H2001-4*** | ICAR-IARI | 0.534 | 0.466 |
|  | **DG(Rg)-29** | ICAR-NBPGR | 0.510 | 0.490 |
|  | **MAL 13*** | ICAR-NRCPB | 0.463 | 0.537 |
|  | **Pusa 992*** | ICAR-IARI | 0.455 | 0.545 |
|  | **EC528322** | ICAR-NBPGR | 0.438 | 0.562 |
|  | **T-10-9** | ICAR-NBPGR | 0.399 | 0.601 |
|  | **Asha*** | ICAR-NRCPB | 0.385 | 0.615 |
|  | **PDF-EE*** | ICAR-IARI | 0.381 | 0.619 |
|  | **PAE-9-569*** | ICAR-IARI | 0.350 | 0.650 |
|  | **DG(Rg)-44** | ICAR-NBPGR | 0.342 | 0.658 |
|  | **AKCMS-13A*** | ICAR-IARI | 0.288 | 0.712 |
|  | **DG(Rg)-16** | ICAR-NBPGR | 0.288 | 0.712 |
|  | **BRG-2*** | ICAR-IARI | 0.287 | 0.713 |
|  | **EC528314** | ICAR-NBPGR | 0.283 | 0.717 |
|  | **ICP15054** | ICAR-NBPGR | 0.280 | 0.720 |
|  | **DG(Rg)-26** | ICAR-NBPGR | 0.273 | 0.727 |
|  | **IC387061** | ICAR-NBPGR | 0.243 | 0.757 |
|  | **DG(Rg)-40** | ICAR-NBPGR | 0.236 | 0.764 |
|  | **ICP15350** | ICAR-NBPGR | 0.187 | 0.813 |
|  | **T-14-18** | ICAR-NBPGR | 0.177 | 0.823 |
|  | **MAL 18*** | ICAR-IARI | 0.139 | 0.861 |
|  | **ICP16675** | ICAR-NBPGR | 0.139 | 0.861 |
|  | **Vipula*** | ICAR-IARI | 0.115 | 0.885 |
|  | **IC33671** | ICAR-NBPGR | 0.088 | 0.912 |
|  | **DG(Rg)-3** | ICAR-NBPGR | 0.074 | 0.926 |
|  | **Pusa 991*** | ICAR-IARI | 0.072 | 0.928 |
|  | **AKCMS-11B*** | ICAR-IARI | 0.068 | 0.932 |
|  | **AKCMS-11A*** | ICAR-IARI | 0.067 | 0.933 |
|  | **U1180** | ICAR-NBPGR | 0.057 | 0.943 |
|  | **ICP15354** | ICAR-NBPGR | 0.050 | 0.950 |
|  | **DG(Rg)-51** | ICAR-NBPGR | 0.043 | 0.957 |
|  | **ICP16451** | ICAR-NBPGR | 0.041 | 0.959 |
|  | **ICP15335** | ICAR-NBPGR | 0.039 | 0.961 |
|  | **DG(Rg)-56** | ICAR-NBPGR | 0.036 | 0.964 |
|  | **ICPL88034*** | ICAR-IARI | 0.034 | 0.966 |
|  | **DG(Rg)-58** | ICAR-NBPGR | 0.032 | 0.968 |
|  | **LRG-141*** | ICAR-IARI | 0.028 | 0.972 |
|  | **EC151154** | ICAR-NBPGR | 0.021 | 0.979 |
|  | **DG(Rg)-39** | ICAR-NBPGR | 0.019 | 0.981 |
|  | **ICP11512** | ICAR-NBPGR | 0.019 | 0.981 |
|  | **DG(Rg)-55** | ICAR-NBPGR | 0.015 | 0.985 |

**Table S7:** Inferred population structure of 95 diverse pigeonpea genptypes used for the development and validation of 62K Axiom SNP chip array based on 33 genome wide unlinked SNPs with minor allele frequencies of ≥0.30. Sub-population I is shaded in red and sub-population II in green with admixure types shown in lighter shades. *Genotypes used for re-sequencing.

| **RIL No.** | **No. of primary branches per plant** | **Pod bearing length main axis (cm)** | **Pod bearing length primary branch (cm)** | **No. of seeds per pod** |
| --- | --- | --- | --- | --- |
| P1 (Pusa Dwarf) | 6.9 | 17.37 | 13.36 | 3.5 |
| P2 (H2001-4) | 8.62 | 27.45 | 19.17 | 3.57 |
| 1 | 4 | 34.25 | 10.4 | 3.44 |
| 2 | 6.5 | 39.6 | 15.8 | 3.62 |
| 3 | 7.3 | 29.1 | 19.8 | 3.1 |
| 4 | 7.8 | 28.2 | 16.9 | 2.92 |
| 5 | 6.7 | 31.3 | 18.65 | 3.5 |
| 6 | 7.77 | 27 | 16.8 | 3.4 |
| 7 | 7.5 | 24.4 | 14 | 3.54 |
| 8 | 7 | 27.4 | 17 | 2.9 |
| 9 | 5.4 | 29.45 | 14.7 | 3.6 |
| 10 | 8.3 | 30.7 | 19 | 3.22 |
| 11 | 10.12 | 32.37 | 14.9 | 3.85 |
| 12 | 9.3 | 32.2 | 21.7 | 17.2 |
| 13 | 9.12 | 28.37 | 14.87 | 3.42 |
| 14 | 7 | 32.1 | 18 | 3.2 |
| 15 | 6.9 | 27.05 | 14.3 | 3.04 |
| 16 | 7.2 | 27.1 | 15.2 | 3.32 |
| 17 | 5.6 | 27.1 | 11.6 | 3.3 |
| 18 | 7.1 | 32.67 | 16.65 | 3.32 |
| 19 | 4.57 | 23.25 | 12.11 | 2.48 |
| 20 | 7.9 | 31.16 | 15.3 | 3.08 |
| 21 | 8.4 | 31.5 | 14.85 | 3.2 |
| 22 | 6.7 | 31.05 | 13.65 | 3.44 |
| 23 | 7.9 | 30.5 | 17.5 | 3.3 |
| 24 | 6.37 | 31.75 | 13.87 | 2.825 |
| 25 | 7 | 30.7 | 13.35 | 3.44 |
| 26 | 6.1 | 32.1 | 17.85 | 3.1 |
| 27 | 7.6 | 25.3 | 14.3 | 3.74 |
| 28 | 7.9 | 29.3 | 14.5 | 2.78 |
| 29 | 12.6 | 29.35 | 17 | 3.46 |
| 30 | 7.9 | 30.1 | 18.9 | 2.84 |
| 31 | 8.7 | 33.2 | 15.15 | 3.54 |
| 32 | 7.8 | 28.8 | 15.7 | 3.42 |
| 33 | 8.1 | 32.9 | 18.95 | 3.22 |
| 34 | 7.6 | 24.95 | 16.8 | 2.88 |
| 35 | 6.3 | 28 | 14.45 | 3.36 |
| 36 | 7.3 | 27.8 | 15.8 | 3.08 |
| 37 | 6 | 27.9 | 15.5 | 3.66 |
| 38 | 8 | 29.4 | 18.4 | 3.24 |
| 39 | 8.8 | 29.2 | 19.7 | 3.84 |
| 40 | 7.2 | 27.2 | 14.5 | 3.9 |
| 41 | 6.7 | 25.8 | 13.8 | 3.14 |
| 42 | 9.6 | 30.95 | 21.3 | 3.74 |
| 43 | 7.2 | 28 | 17.5 | 3.7 |
| 44 | 8.6 | 27 | 12.8 | 3.56 |
| 45 | 6.5 | 31.3 | 14.9 | 3.6 |
| 46 | 5.7 | 23.8 | 13.95 | 3 |
| 47 | 8.3 | 26.9 | 15.4 | 4 |
| 48 | 7.9 | 30.6 | 18.65 | 3.82 |
| 49 | 5.1 | 8.5 | 6.7 | 3.86 |
| 50 | 7.5 | 27.4 | 15.6 | 3.58 |
| 51 | 10.4 | 33.5 | 19.3 | 3.66 |
| 52 | 8.9 | 30.9 | 18.3 | 3.58 |
| 53 | 7.5 | 29.15 | 16.8 | 3.1 |
| 54 | 8.9 | 28.75 | 18.1 | 3.5 |
| 55 | 7.4 | 29.4 | 17.75 | 3.52 |
| 56 | 7 | 30.9 | 19.5 | 3.44 |
| 57 | 8.5 | 33.6 | 17.45 | 3.38 |
| 58 | 6.3 | 32.1 | 18.5 | 3.5 |
| 59 | 6.3 | 26.5 | 18.1 | 2.72 |
| 60 | 6.3 | 25.3 | 13.9 | 3.14 |
| 61 | 9.7 | 27.1 | 17.7 | 3.46 |
| 62 | 7.8 | 30 | 20 | 3.66 |
| 63 | 7.9 | 30.3 | 16.75 | 3.76 |
| 64 | 7.8 | 28.85 | 18.8 | 3.64 |
| 65 | 5.2 | 19.6 | 12.6 | 3.64 |
| 66 | 7.5 | 24.5 | 17.5 | 2.48 |

**Table S8:**  Mean phenotypic data on RIL mapping population and parents (Pusa Dwarf/H2001-4) for four yield related traits of pigeonpea.
